# Intestinal Bacterial Communities of Trypanosome-Infected and Uninfected *Glossina palpalis* palpalis from Three Human African Trypanomiasis Foci in Cameroon

**DOI:** 10.3389/fmicb.2017.01464

**Published:** 2017-08-03

**Authors:** Franck Jacob, Trésor T. Melachio, Guy R. Njitchouang, Geoffrey Gimonneau, Flobert Njiokou, Luc Abate, Richard Christen, Julie Reveillaud, Anne Geiger

**Affiliations:** ^1^UMR INTERTRYP, Institut de Recherche pour le Développement-CIRAD, CIRAD TA A-17/G Montpellier, France; ^2^Parasitology and Ecology Laboratory, Department of Animal Biology and Physiology, Faculty of Science, University of Yaounde 1 Yaounde, Cameroon; ^3^UMR MIVEGEC, Institut de Recherche pour le Développement 224-Centre National de la Recherche Scientifique 5290 Montpellier, France; ^4^UMR 7138, Systématique Adaptation Evolution, Université de Nice-Sophia Antipolis Nice, France; ^5^Institut National de la Recherche Agronomique, UMR 1309 ASTRE Montpellier, France; ^6^CIRAD, UMR ASTRE Montpellier, France

**Keywords:** *Glossina*, trypanosome, microbiome, meta-taxonomics, sleeping sickness, nagana

## Abstract

*Glossina* sp. the tsetse fly that transmits trypanosomes causing the Human or the Animal African Trypanosomiasis (HAT or AAT) can harbor symbiotic bacteria that are known to play a crucial role in the fly's vector competence. We hypothesized that other bacteria could be present, and that some of them could also influence the fly's vector competence. In this context the objectives of our work were: (a) to characterize the bacteria that compose the *G. palpalis palpalis* midgut bacteriome, (b) to evidence possible bacterial community differences between trypanosome-infected and non-infected fly individuals from a given AAT and HAT focus or from different foci using barcoded Illumina sequencing of the hypervariable V3-V4 region of the *16S rRNA gene*. Forty *G. p. palpalis* flies, either infected by *Trypanosoma congolense* or uninfected were sampled from three trypanosomiasis foci in Cameroon. A total of 143 OTUs were detected in the midgut samples. Most taxa were identified at the genus level, nearly 50% at the species level; they belonged to 83 genera principally within the phyla Actinobacteria, Bacteroidetes, Firmicutes, and Proteobacteria. Prominent representatives included *Wigglesworthia* (the fly's obligate symbiont), *Serratia*, and *Enterobacter hormaechei. Wolbachia* was identified for the first time in *G. p. palpalis*. The average number of bacterial species per tsetse sample was not significantly different regarding the fly infection status, and the hierarchical analysis based on the differences in bacterial community structure did not provide a clear clustering between infected and non-infected flies. Finally, the most important result was the evidence of the overall very large diversity of intestinal bacteria which, except for *Wigglesworthia*, were unevenly distributed over the sampled flies regardless of their geographic origin and their trypanosome infection status.

## Introduction

Animal African Trypanosomiasis (AAT), or Nagana, is primarily caused by *Trypanosoma brucei brucei* (Tbb), *Trypanosoma vivax*, and *Trypanosoma congolense*, which are transmitted to their mammalian hosts by tsetse flies belonging to the species *Glossina palpalis* and *Glossina morsitans*. This disease is responsible for significant economic losses in areas where cattle are exposed to parasites (Shaw et al., 2013). Human African Trypanosomiasis (HAT), or sleeping sickness, is endemic to Sub-Saharan Africa, where it causes significant health and economic impacts (Welburn and Maudlin, 2012). The chronic form of HAT is caused by *Trypanosoma brucei gambiense* (Tbg), whereas *Trypanosoma brucei rhodesiense* (Tbr) causes the acute form; the two forms are transmitted to humans by *G. palpalis* and *G. morsitans*, respectively.

Currently, about 60 million people continue to be at risk for HAT in 36 Sub-Saharan African countries. Several factors complicate the treatment of HAT. The medications that are currently used to treat human patients can generate many side effects (reviewed in Geiger et al., 2011a). In addition, the emergence of drug-resistant trypanosomes was recently reported (Baker et al., 2013). These factors are pertinent, since epidemiological modeling of the impact of global change on HAT expansion predicts that 46–70 million more people will be at risk by 2090 (Moore et al., 2012). Thus, the search for new control strategies is a priority to impede the spread of the disease. In this context, identifying the factors involved in fly vector competence (as well as understanding how they work) may open new perspectives.

Trypanosomes ingested by the tsetse fly during a blood meal on an infected host passively reach the tsetse intestine, where they differentiate into their procyclic form, mature, and multiply. Then, depending on the species, the trypanosomes migrate toward either the fly's salivary glands (in the case of *T. brucei* sp.) or the mouth parts, where they undergo the maturation step into their infective metacyclic form. The trypanosomes may then be transmitted to a mammalian host during a subsequent blood meal ingested by the fly (Vickerman et al., 1988). This ability of tsetse flies to acquire trypanosomes and to subsequently favor their maturation and transmit them to a vertebrate host, known as the fly's vector competence, depends on several factors including the production of antimicrobial peptides (e.g., cecropin and attacin; Hu and Aksoy, 2006), reactive oxygen species (MacLeod et al., 2007), EP-protein [Glutamic acid (E)—Proline (P) rich protein, an immune responsive protein], (Haines et al., 2010), intestinal lectin (Welburn et al., 1994), as well as the peritrophic matrix (Weiss et al., 2013, 2014; Rose et al., 2014; Aksoy et al., 2016).

As previously reported, tsetse flies can harbor three different symbionts that play a crucial role in the physiology of their host. First, the obligate Enterobacteria *Wigglesworthia glossinidia* is present intracellularly in the bacteriocytes, specialized cells of the intestine that form the bacteriome (Aksoy, 1995). This ancient host association (Chen et al., 1999) is involved in the tsetse fly's fertility, nutrition, and the development of its immune system (Akman et al., 2002; Weiss et al., 2011; Rio et al., 2012). Second, the facultative symbiont *Sodalis glossinidius* is present in the cells of the intestine, but it can also be found extracellularly and in other tissues of the fly (Cheng and Aksoy, 1999; Balmand et al., 2013). There is substantial evidence that certain *S. glossinidius* genotypes favor the establishment of trypanosomes in the fly's gut (Geiger et al., 2007; Farikou et al., 2010a; Hamidou Soumana et al., 2014). *W. glossinidia* and *S. glossinidius* are both transmitted maternally to the progeny *via* milk secretions during the intra-uterine development of the larvae (Aksoy, 1995). Third, the facultative symbiont *Wolbachia* can be detected very early in oocytes, embryos and larvae (Cheng et al., 2000). This symbiont acts on the reproductive process of tsetse flies by inducing cytoplasmic incompatibility (Alam et al., 2011). *Wolbachia* infection has been negatively correlated with the prevalence of trypanosome and of salivary gland hypertrophy virus (Alam et al., 2012). This indicates that *Wolbachia* may have a role in preventing infection of tsetse flies from trypanosomes (Aksoy et al., 2013), which interestingly has not been revealed for plasmodium infection in mosquitoes that harbor *Wolbachia* (Zélé et al., 2014). This symbiont is trans-ovary transmitted to the progeny (Cheng and Aksoy, 1999).

The presence of bacteria (other than symbionts) in the gut of field-collected tsetse flies has been assessed by bacterial isolation and culture approaches (Geiger et al., 2009, 2011b; Lindh and Lehane, 2011), as well as by direct molecular identification methods. Using deep sequencing of the V4 hypervariable region of the *16S rRNA* gene, Aksoy et al. (2014) revealed the limited diversity of gut-specific microbiota in wild tsetse flies from Uganda. Surprisingly, bacteria belonging to the genera *Enterobacter, Acinetobacter*, and *Enterococcus* were found in the gut of the blood-sucking fly, despite the fly feeds on animals which blood is normally devoid of any bacteria. The source of these bacteria is unknown, but their presence could be due to the fact that tsetse flies can occasionally feed on nutrients other than blood that may be contaminated by diverse bacteria (Colman et al., 2012). Flies may also ingest bacteria present on the skin surface of the hosts when taking their bloodmeal (Poinar et al., 1979). Finally, differences in their diet that may depend on the environmental conditions could lead to differences in the fly's gut bacteriome as it was shown for other insects (Colman et al., 2012). Finding such bacteria in an organ where trypanosomes are localized raised the question whether these bacteria could be involved, besides the known symbionts, in fly infection by trypanosomes, inasmuch as certain bacteria species have been shown to produce antiparasitic molecules (Lazaro et al., 2002; Moss, 2002). In this context, a novel bacterial species, *Serratia glossinae*, has been identified in the gut of insects reared in insectarium (Geiger et al., 2010). This bacterium is related to *Serratia marcescens* (Enterobacteriaceae) a bacterium involved in the production of pigments that are toxic for *Trypanosoma cruzi* (Azambuja et al., 2004). In *Anopheles*, the abundance of intestinal Enterobacteriaceae has been associated to the presence of the parasite *Plasmodium falciparum* (Boissière et al., 2012). This illustrates the need for a better understanding of the composition of the tsetse fly bacteriome and its potential variations, since differences in the environment could induce differences in the bacteriome composition which, in turn, could induce differences in the fly vector competence.

Using bar-coded Illumina sequencing of the hypervariable V3-V4 region of the *16S rRNA* gene, we aimed to characterize bacterial communities in the midgut of *G. p. palpalis* sampled from three different sleeping sickness foci in Cameroon, and to verify the hypotheses that the composition of the midgut bacterial communities of trypanosome-infected *G. p. palpalis* might be different from that of non-infected flies across these three HAT foci. Finally we aimed to identify bacteria that could be associated with the infection status of the fly. Those, if any, associated with fly refractoriness would be of particular interest inasmuch if they would be maternally transmitted to offspring. In the case they would not be maternally transferred, their trypanocidal compound(s) should be identified and possibly delivered via the paratransgenic approach described by Rio et al. (2004). Both approaches may offer the possibility to block the fly vector competence, hence the spread of the sleeping disease.

## Materials and methods

### Sampling areas

**Figure 1:**
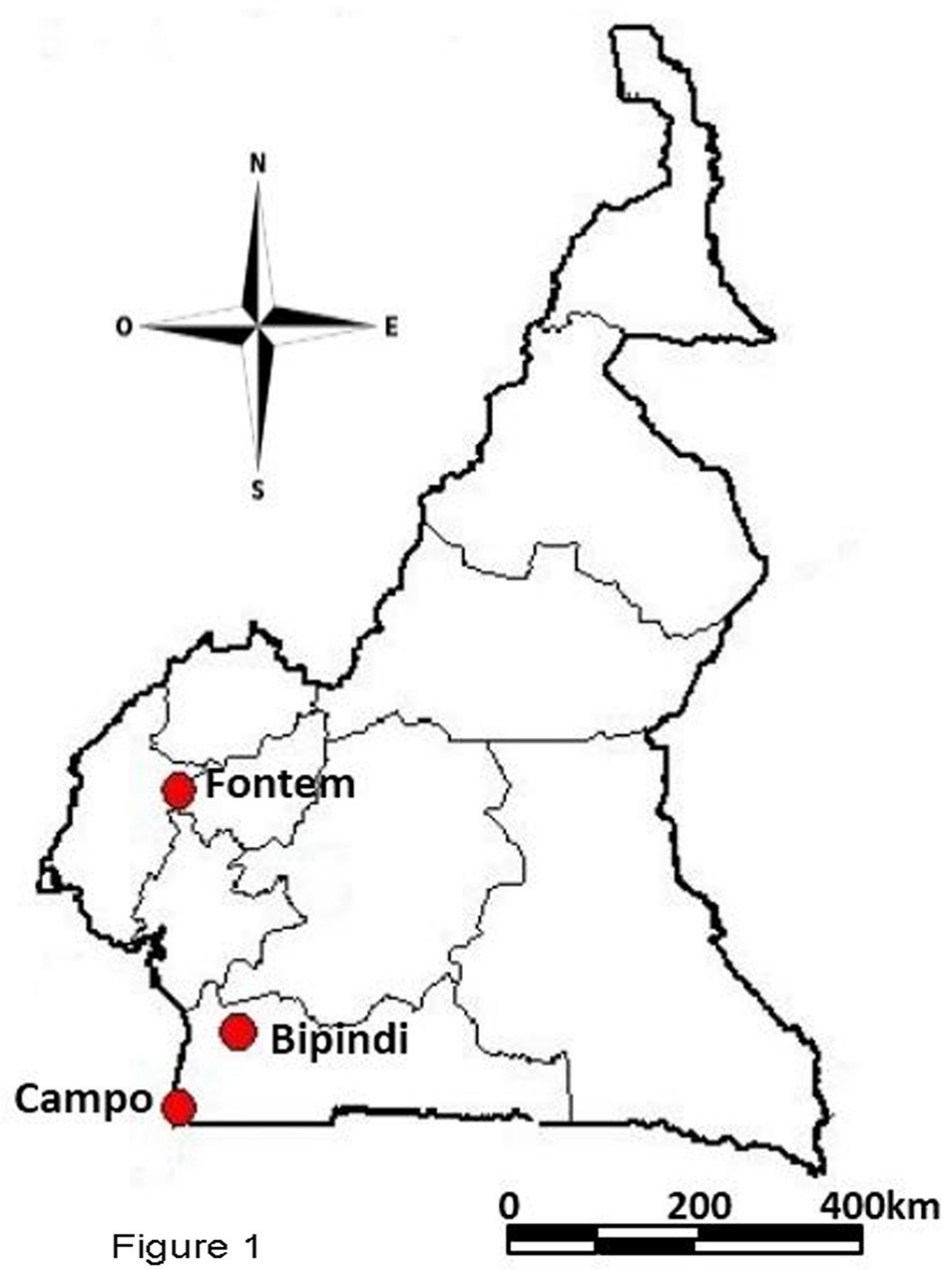
Geographical map showing the three described Cameroonian HAT foci.

The study was performed in three sleeping sickness foci (Bipindi, Campo, and Fontem) located in the forest region of southern-Cameroon (Figure 1).

Bipindi (3°2′N, 10°22′E) is a historical sleeping sickness focus originally described in 1920 (Grébaut et al., 2001). It is situated between Lolodorf and Kribi, 75 km from the Atlantic Ocean. Although, a 2004 survey identified four tsetse fly species (Grébaut et al., 2004), *G. p. palpalis* was the only species found in this focus since 2007 (Farikou et al., 2010a,b; Melachio et al., 2011; Tchouomene-Labou et al., 2014). Bipindi is surrounded by hills and covers several villages, mainly located along the roads. The focus has a typical forest bioecological environment, including equatorial forest and farmland along the roads and surrounding the villages. Bipindi is home to highly diverse wild fauna (Njiokou et al., 2004, 2006) and it has a dense network of rivers crossing cocoa farms, all of which offer suitable habitats for tsetse flies.

Campo (2°20′N, 9°52′E) is a hypo-endemic focus where several cases of sleeping sickness are diagnosed every year. It is located along the Ntem River, which separates Cameroon from Equatorial Guinea. Several tsetse fly species (including *G. p. palpalis*, and to a lesser extent *G. pallicera, G. caliginea*, and *G. nigrofusca*) can be encountered in this focus (Simo et al., 2008). Several biotopes are found in the Campo focus, including farmland, marshes, swampy areas, and rainy forest.

Fontem (5°40′N, 9°55′E), in the Southwest Region of Cameroon, has been recognized as a sleeping sickness focus since 1949. Since that time, the prevalence of HAT has decreased considerably. The landscape is characterized by the presence of hills and valleys that are crossed by rivers with fast currents. *G. p. palpalis* is the only tsetse fly species found in this area (Simo et al., 2008). The focus is divided into the North, Center and South sub-foci. The Center sub-focus (comprising the villages of Menji, Fotabong, Nsoko, and Azi), where flies were sampled, has only had a few sleeping sickness cases in the past decade. In the Northern sub-focus (comprising the villages of Bechati, Folepi, and Besali) no new HAT patients have been diagnosed in the last 20 years (Ebo'o Eyenga, personal communication), although 15% of all pigs were infected with *T. b. gambiense* group 1 in 1999 (Nkinin et al., 2002).

### Fly sampling and experimental design

Entomological surveys were conducted in the Campo focus in March 2008, and in the Bipindi and Fontem foci in November 2013. Pyramidal traps (Gouteux and Lancien, 1986) were placed for 4 consecutive days in suitable tsetse fly biotopes of each village located within each focus. The geographical position of the sampling sites was determined by GPS. Trapped tsetse flies were harvested twice a day (before 12 a.m. and before 3 p.m.).

The *Glossina* species were first identified according to morphological criteria (Grébaut et al., 2004), and then sorted into two groups of teneral (young flies that had never taken a blood meal) and non-teneral flies, respectively. Among the 599 non-teneral flies that were caught in the Campo focus, 200 were randomly sampled, their midguts dissected (see below) and observed under a light microscope (magnification x100) to detect the presence of motile trypanosomes (infected flies) or the absence of trypanosomes (non-infected flies). The trypanosomes from infected flies were subsequently identified (species or sub-species) by PCR using specific primers (Table 1). The absence of any trypanosomes (negative PCR assay) was confirmed in all non-infected flies using the same specific primers. Among the 300 flies caught in Bipindi, 200 were sampled and processed as for the Campo flies. Only 27 non-teneral flies could be caught in Fontem; as shown after dissection, microscopy, and PCR analyses, they all were non-infected by trypanosomes.

**Table 1:** Primers used for PCR amplification of trypanosome DNA (Farikou et al., 2010a).

**Specificity**	**Primer sequence**	**Amplified product (bp)**	**References**
*T. congolense* (forest type)	5′-GGACACGCCAGAAGGTACTT-3′	350	Masiga et al., 1992
	5′-GTTCTCGCACCAAATCCAAC-3′		
*T. congolense* (savannah type)	5′-TCGAGCGAGAACGGGCACTTTGCGA-3′	341	Moser et al., 1989
	5′-ATTAGGGACAAACAAATCCCGCACA-3′		
*T. brucei* s.l.	5′-CGAATGAATATTAAACAATGCGCAG-3′	164	Masiga et al., 1992
	5′-AGAACCATTTATTAGCTTTGTTGC-3′		
*T. vivax*	5′-CGACTCCGGGCGACCGT-3′	600	Majiwa et al., 1994
	5′-CATGCGGCGGACCGTGG-3′		

**Figure 2:**
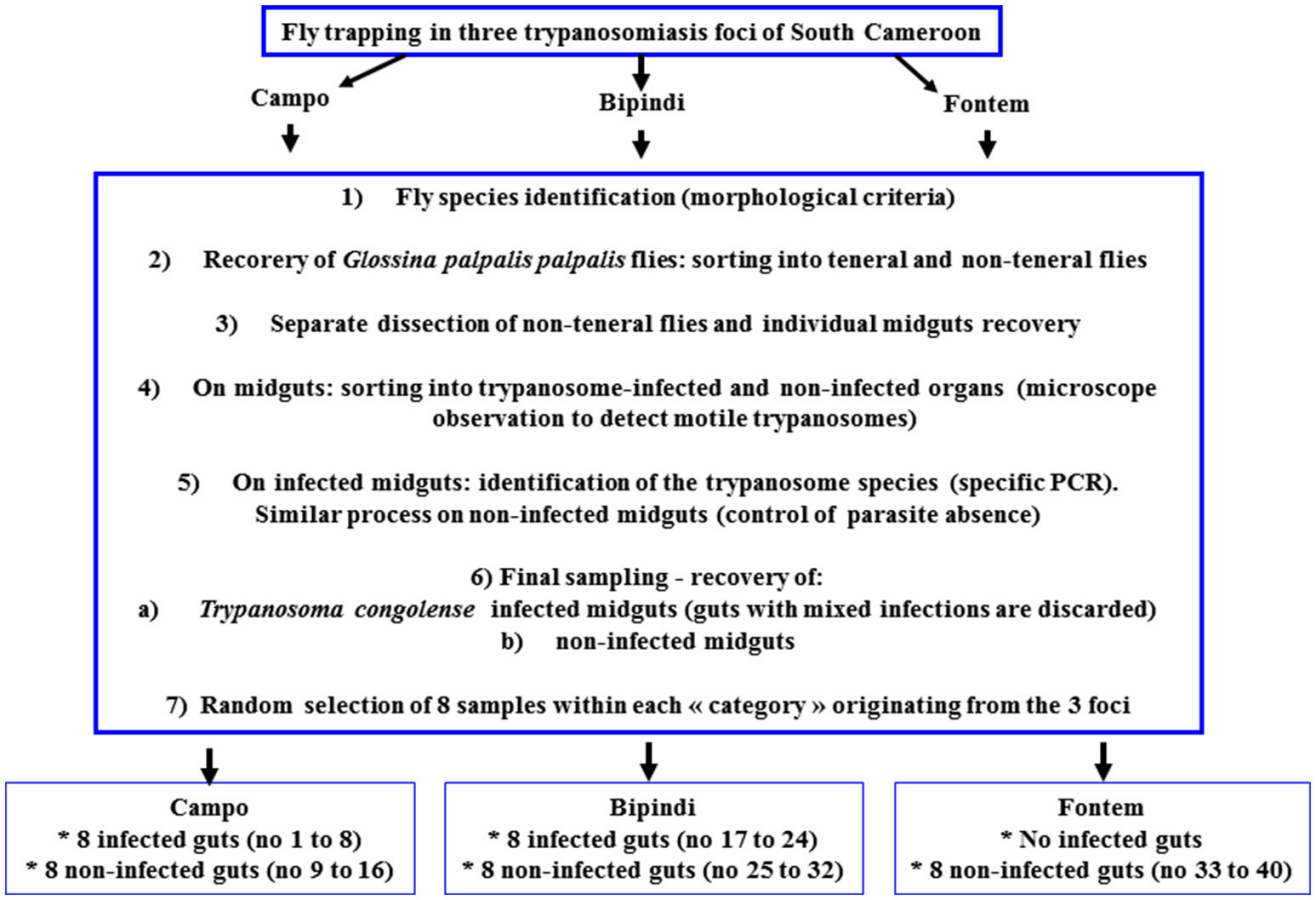
*Glossina* sampling and selection process.

The microbiota analysis was performed on *G. p. palpalis* flies that had undergone further selection. The midguts from the 200 sampled flies from Campo were separated into two groups composed, respectively, of trypanosome infected and non-infected midguts. Among the infected midguts, only those harboring *T. congolense* (either the forest or savannah type; mixed infections were discarded) were taken into account. Finally, from the non-infected group and from the *T. congolense* infected group, respectively, eight midguts were randomly selected and identified by numbering. The 200 samples from Bipindi were processed similarly. Among the midguts from the 27 flies from Fontem, eight were randomly selected, which formed a unique group of non-infected samples. In total, five groups were formed that differed from each other regarding the infection status of the flies and/or their geographic origin. The “sex ratio” (12.5, 37.5, 37.5, 75, and 25% male flies in, respectively group 1–5) was recorded but not taken into account in the subsequent analyses. The characteristics of each group, including sample numbering, are the followings: “Group 1/Campo infected flies: number 1–8; Group 2/Campo non-infected flies: number 9–16; Group 3/Bipindi infected flies: number 17–24; Group 4/Bipindi non-infected flies: number 25–32; Group5/Fontem non-infected flies: number 33–40 (Figure 2).

### Dissection of tsetse flies, DNA extraction, and trypanosome PCR amplification

The non-teneral flies were surface-sterilized (once with 5% sodium hypochlorite for 10 min and twice with 70% ethanol, each for 10 min) and the midgut of each fly was dissected under sterile conditions using a Bunsen burner, and in sterile NaCl 0.9%. The instruments used were carefully cleaned after the dissection of each fly to prevent cross-contamination. Midguts were recovered and then separately transferred into tubes containing 95° ethanol for further DNA extraction. During the field surveys (10 days for each mission), tubes containing the samples were maintained at −20°C; thereafter, in the laboratory, the midgut samples were immediately processed and subjected to DNA extraction using the DNeasy kit (Qiagen, Paris, France) according to Geiger et al. (2007). Genomic DNA was quantified using NanoDrop (Thermo Scientific, Paris).

PCR amplification was performed as previously described (Farikou et al., 2010a) using trypanosome-specific primers (Table 1; Moser et al., 1989; Masiga et al., 1992; Majiwa et al., 1994; Herder et al., 2002). This procedure allowed detection of *T. brucei s.l., T. congolense* (both forest and savannah types), and *T. vivax*.

### Meta-barcoding analysis

Amplification of the highly variable V3-V4 region of the *16S rRNA* gene, using the forward primer 5′-CCTACGGGNGGCWGCAG-3′ and the reverse primer 5′-GACTACHVGGGTHTCTAATCC-3′ (N representing A, C, G, or T; V representing A, C, or G; H representing A, C, or T; W representing A or T; IUPAC codes), was performed to evaluate the bacterial communities of tsetse midguts on the Illumina MiSeq (MR DNA Laboratory-http://www.mrdnalab.com/shallowater, USA). The primers were specifically designed by one of the co-authors of the current study. As previously described in the method of Porter et al. (2016), each PCR amplifying medium contained 2 μL DNA template, 17.5 μL molecular biology-grade water, 2.5 μL 10× reaction buffer, 1 μL 50× MgCl2 (50 mM), 0.5 μL dNTPs mix (10 mM), 0.5 μL forward primer (10 mM), 0.5 μL reverse primer (10 mM), and 0.5 μL Invitrogen Platinum *Taq* polymerase (5 U/μL) in a total volume of 25 μL. The amplification program was: 95°C for 5 min, followed by 25 cycles of 94°C for 40 s, 55°C for 1 min, and 72°C for 30 s, and lasted at 72°C for 5 min. Amplicons were purified using MiniElute PCR purification columns (Qiagen, Paris, France) and were eluted in 50 μL molecular biology-grade water. The purified amplicons from the first PCR round served as templates for the second PCR round, using Illumina adaptor-tailed primers in a 10-cycle amplification regime. All PCR reactions were performed in an Eppendorf Mastercycler ep gradient S thermal cycler. Negative controls (containing all reagents except the midgut DNA that was replaced by water) were included in all experiments. Amplicons were quantified using a Bioanalyzer (Agilent Technologies, Paris) and then the KAPA Quantification Kit (Roche, Paris). Equimolar amounts of the generated libraries were dual-indexed, combined, and sequenced on an Illumina MiSeq platform (MRDNA Laboratory, Shallowater, USA) using MiSeq Reagent kits (300 cycles), following the 2 × 300-bp paired-end sequencing protocol. The generated sequences have been deposited in the EMBL-EBI (Study accession number PRJEB14010; Secondary study accession number ERP015605 and Locus tag prefix BN6929).

### Quality filtering and taxonomic classification of sequencing data

Illumina MiSeq reads were analyzed using in-house pipelines (Richard Christen; described in Boissière et al., 2012; Hartmann et al., 2012; Gimonneau et al., 2014; Massana et al., 2015). Briefly, Silva 119 NR (analyses performed in 2014) was used as the reference database for taxonomic identification (Quast et al., 2012). An *in silico* extraction of Silva amplicons using forward: 5′-CCTACGGGNGGCWGCAG-3′ and reverse: 5′-GACTACHVGGGTHTCTAATCC-3′ primers, followed by an analysis by length/number of amplicons, yielded the following results: 1–50/0; 51–100/0; 101–150/2; 151–200/1; 201–250/1; 251–300/11; 301–350/40; 351–400/21,669; 401–450/448,575; 451–500/450; 501–550/192; 551–600/1,198; 601–650/0. This demonstrates that most amplicons were between 350 and 450 nucleotides in length. The extracted database is referenced below as the refseq. Each sequence identifier was reformatted to eight taxonomic fields as in PR2 (Guillou et al., 2012) making it easier to use a pipeline for analyses.

In order to assemble paired-end reads, the software programs PEAR (Zhang et al., 2014) and FLASH (Magoc and Salzberg, 2011) were both tested. PEAR using default parameters merged more pairs, and paired-end reads were therefore assembled and quality filtered (using Illumina quality scores) with PEAR. 94.26% (3,622,233 reads) of the total reads (3,842,672 reads) were assembled; 0.003% were discarded and 5.734% were not assembled.

Reads were then sorted by length using a dedicated python script, yielding the following results: 0–0/0; 1–50/3; 51–100/1,556; 101–150/1,435; 151–200/1,535; 201–250/1,578; 251–300/2,251; 301–350/9,401; 351–400/38,323; 401–450/132,487; 451–500/3,433,239; 501–550/381; 551–600/44; 601–650/0. Reads shorter than 300 nucleotides (0.23% out of the overall number of reads) were discarded, resulting in the extraction of 3,613,875 reads. The fasta file was then demultiplexed using a dedicated C++ program. Primer trimming was performed using CutAdapt v1.8.1 (Martin, 2011).

Each file was dereplicated, sorted by decreasing abundance, chimera checked with UCHIME (Edgar et al., 2011) and then clustered using Crunchclust (Mondani et al., 2011; Gimonneau et al., 2014; Massana et al., 2015; Tchioffo et al., 2016; available from https://github.com/dumaatravaie/crunchclust/blob/master/Documentation; at a Levenstein distance of 5). After this clustering step, clusters that contained <2 reads were discarded as artifacts (see Boissière et al., 2012). All these steps resulted in 2,562,144 high-quality sequences contained in clusters with at least two reads (70.73% of the total assembled reads) that were subsequently used for taxonomic assignment. In each cluster, the most abundant sequence was kept as the representative one, since it was assumed to have the least errors in a cluster. Taxonomic assignment was done as in Pawlowski et al. (2011). Briefly, a Needleman–Wunsch algorithm to search for the 30 most similar sequences to each representative sequence from the refseq was employed. The reference sequences with the highest percentage were then used, and taxonomy to a given level was obtained. When more than one result emerged, the two highest hits were reported. When similarity was <80%, sequences were not assigned. Abundance matrices were generated for statistical analyses at each taxonomic level. Several abundant Operational Taxonomic Units (OTUs) could not be identified satisfactorily down to the genus or species level. In these cases (rough estimation: 1%), reads sequences and similar refseq sequences were selected and then aligned using ClustalO (Sievers et al., 2011) and SeaView (Gouy et al., 2010). Trees were plotted using TreeDyn (Chevenet et al., 2006) or MetaPhlAn (Segata et al., 2012), and distinct robust subtrees were annotated as distinct species whenever possible.

### Data and statistical analysis

Statistical analyses were performed using the R package vegan. Rarefaction curves (Figure S1) were performed prior to comparative analyses between infected and uninfected flies, and between the sampling sites. Significant differences in bacterial richness between the infected and uninfected flies, and between the three sampling sites were tested using non-parametric Kruskal–Wallis test. We used vegdist and hclust using single, average, and complete linkage methods for hierarchical clustering and then compared them for the presence of sub-trees. Nonmetric multidimensional scaling (nMDS) were generated using the R packages ggplot2 and phyloseq (McMurdie and Holmes, 2013).

## Results

### Distribution of tsetse flies by infected vs. non-infected status

**Figure 3:**
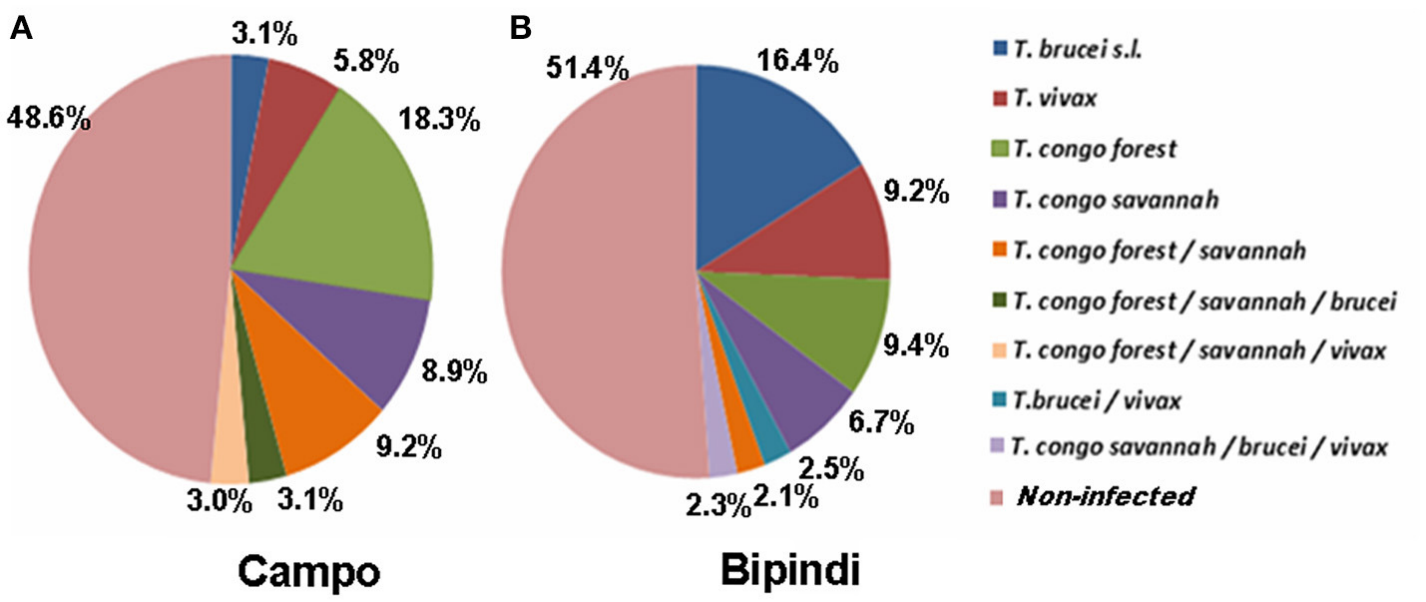
Prevalence of the different trypanosome species or subspecies infecting tsetse flies collected in the Campo **(A)** and Bipindi **(B)** foci. Single as well as mixed infections have been considered.

The distribution of trypanosome species and/or subspecies in the midgut of tsetse flies from Campo (200 flies) and Bipindi (200 flies) is displayed in Figure 3. Nearly half of all flies, whether from Campo (51.4%) or from Bipindi (48.6%), were infected with at least one of the four studied trypanosome species and/or subspecies. The prevalence of the four trypanosome species was highly dependent on the sampling site. In flies sampled from Campo, *T. congolense* (both forest and savannah types) displayed the highest prevalence, accounting for 83% of all infected flies (including mixed infections), while *T. brucei sensu lato* displayed the lowest prevalence (12%, including mixed infections). In contrast, *T. brucei s*.*l*. displayed the highest prevalence (43%) in infected flies from Bipindi, with *T. congolense* accounting for 42%. As for the Fontem focus, the low number of collected flies (27 flies) prevented any reliable statistical analysis regarding the prevalence of the infected flies in this focus, inasmuch as all of the 27 flies were uninfected (which does not imply that the overall population of flies is uninfected).

### Bacterial communities in the G. *p. palpalis* midgut

**Figure 4:**
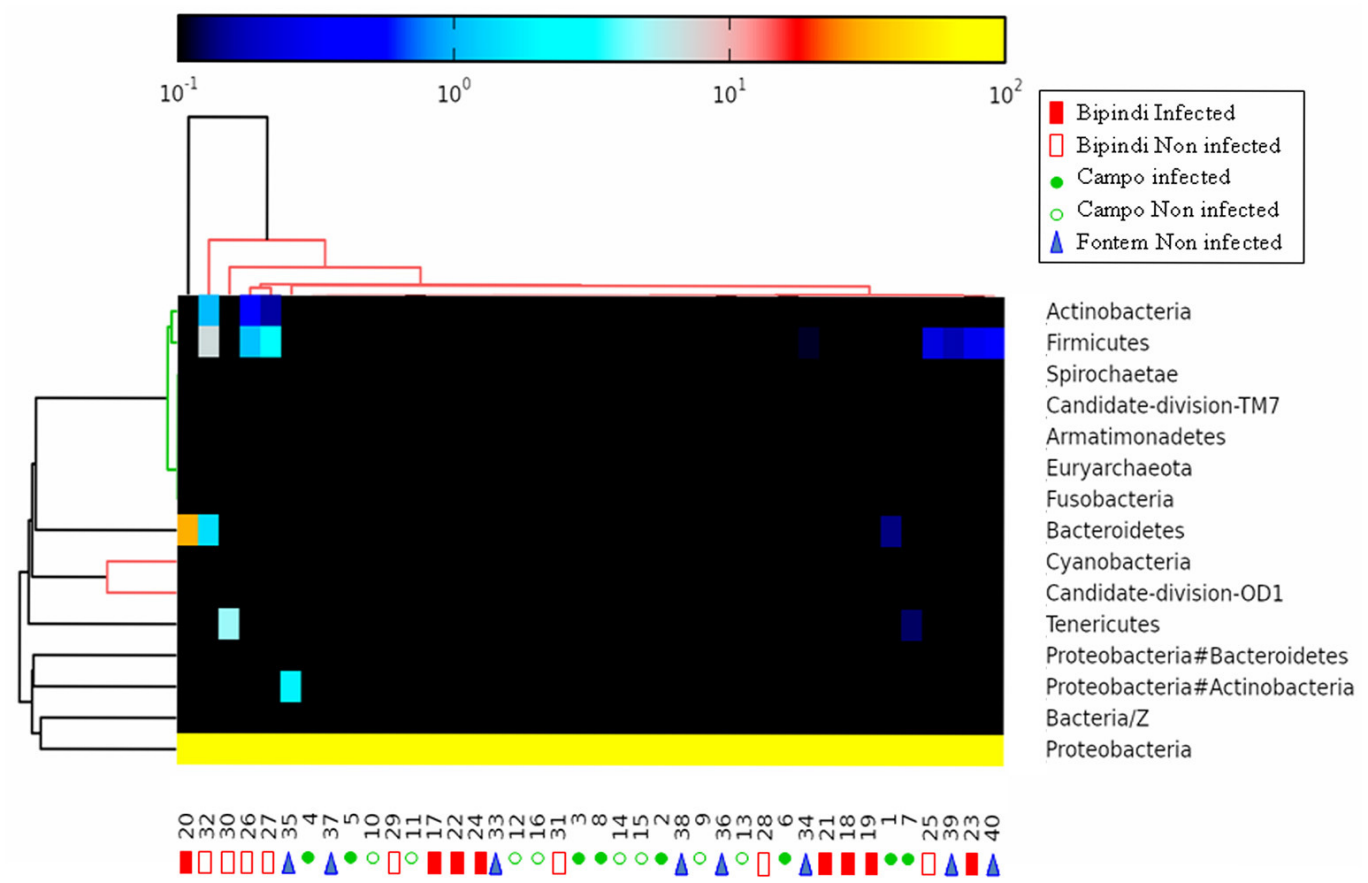
Heat map analysis of the distribution and abundance of the bacterial phyla in midgut samples. The samples did not cluster together, indicating highly variable bacterial communities. Numbers 1–8: infected samples from the Campo focus; Numbers 9–16: non-infected samples from the Campo focus; Numbers 17–24: infected samples from the Bipindi focus; Numbers 25–32: non-infected samples from the Bipindi focus; Numbers 33–40: non infected samples from the Fontem focus.

Illumina sequencing of *16S rDNA* gene generated a total of 2,562,144 high-quality sequence reads across the V3–V4 region in 40 tsetse flies, for the 40 samples randomly selected. The average number of tags/sample for the V3–V4 region was 12,988 (ranging from 6,525 to 19,694 per gut), with read length varying from 300 to 600 nucleotides. After taxonomic assignation, a total of 13 phyla were identified from the 40 gut samples (Table S1); 12 phyla belonged to the Bacterial kingdom and one phylum belonged to the Archaea. Proteobacteria were present in all 40 samples and had a relative abundance higher than 68% in each sample (even higher than 97% in 37 out of 40 samples), while the other phyla (as well as their respective relative abundances) were unevenly distributed among the different samples. The Bacteroidetes phylum was detected in 26 samples, Firmicutes was observed in 19 samples, Proteobacteria/Actinobacteria was detected in 14 samples, and Actinobacteria was found in 7 samples. The 7 other phyla could only be detected in 4 samples or less. Samples from groups 1 and 2 (infected and non-infected midguts from Campo; sample numbers 1–16) harbored 1 to 4 different phyla; samples from group 3 (infected midguts from Bipindi; sample numbers 17–24) contained 2–5 different phyla; samples from group 4 (non-infected midguts from Bipindi; sample numbers 25–32) contained 3–7 different phyla; and samples from group 5 (non-infected midguts from Fontem; sample numbers 33–40) harbored 1–4 different phyla (Table S1). A heat map analysis of the distribution and abundance of the bacterial phyla showed the sampled midgut did not cluster together, confirming that bacterial communities at the phylum level were unevenly distributed among the samples (Figure 4).

The distribution of bacterial taxa among the 40 samples at the class and genus levels and their relative abundance in each sample are reported in Table S1. Twenty-four classes and 83 genera could be distinguished. The gut microbiota presented a large inter-individual variability, although only a few taxa were prominent.

#### Bacteria classes

**Figure 5:**
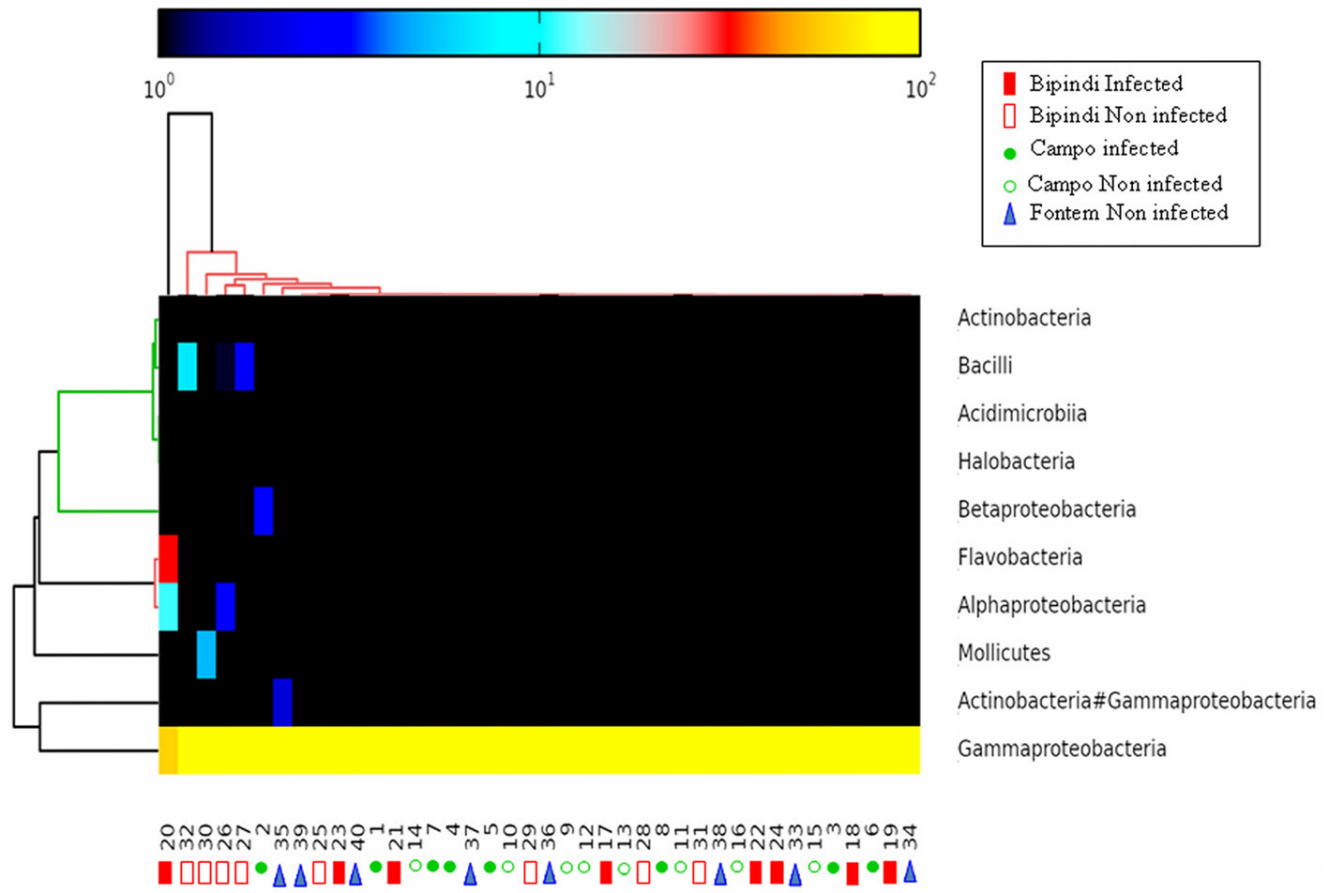
Heat map analysis of the distribution and abundance of the bacterial classes in midgut samples. The samples did not cluster together, indicating highly variable bacterial communities. Sample numbering as in Figure 4.

As reported above, all 40 samples contained Proteobacteria, accounting for more than 97% of all microbiota in 37 samples. The Gammaproteobacteria, present in all 40 samples and accounting for more than 95% of the total microbiota in 38 samples, was the most prominent class in this phylum. Alpha-proteobacteria (maximal abundance per sample: 11.08%) were found in 32 samples. In contrast, Beta-proteobacteria were present in only 7 samples (maximal abundance per sample: 2.38%). Flavobacteria were present in 26 samples (maximum abundance per sample: 31.09%). Bacteroidia, Mollicutes, Actinobacteria/Gammaproteobacteria, and Bacilli were present in 1, 4, 14, and 19 samples, respectively [maximum abundance (%) of the total microbiota per sample: 0.6, 4.96, 1.88, and 4.11, respectively]. Finally, Actinobacteria were present in 7 samples (maximum abundance per sample: 0.19%). The heat map analysis in Figure 5 shows the distribution of the most abundant classes (those that were present in at least 7 samples) among the different samples. The other identified classes, such as Negativicutes, Rubrobacteria, Sphingobacteriia, and Clostridia, were present in <7 samples at a frequency of <0.25% per sample. In general, bacterial classes were very unevenly distributed among the samples. For example, whereas the abundance of Gammaproteobacteria was 100% in sample 15 and sample 32, sample 20 harbored representatives of Gammaproteobacteria, Flavobacteria, and Alphaproteobacteria (respective abundance: 57.74, 31.11, and 11.10%).

#### Bacterial genera

Out of the 40 samples, 83 different bacteria genera (Table S1) were detected. The heat map analysis (Figure S2) performed on genera present in at least four samples, displays the distribution of genera, and their respective abundance, among the 40 samples. Midgut bacteria were unevenly distributed among individual tsetse flies and between the different sampled localities. Several genera were found in all (or most of) tsetse flies, possibly representing the midgut core microbiota. This was the case for the genus *Wigglesworthia*, as well as for several genera belonging to the Enterobacteriaceae family. The relative abundance of *Wigglesworthia* ranged from 34.5 to 99.8% across the 40 samples. Other genera widely distributed included two *Enterobacter* that were present, respectively, in 40 (relative abundance: 0.02–61.1%) and in 21 samples (relative abundance: 0.02–1.11%); *Candidatus Sulcia muelleri*, present in 26 samples (relative abundance: 0.01–31.09%); two *Serratia* present in 29 samples (relative abundance: 0.01–14%) and in 20 samples (relative abundance: 0.01–4.95%). Furthermore, *Wolbachia* was present in 25 samples (relative abundance: 0.01–11.08%), while *Sodalis* was only present in 4 samples, with a maximum relative abundance of 0.06%.

These results indicate that the midgut bacterial flora was mainly composed of gram-negative communities. Gram-positive bacteria belonged to the Bacilli, Mollicutes, Actinobacteria, Rubrobacteria, Clostridia, Fusobacteriia, Acidimicrobiia, and Thermoleophilia classes, and represented 10% of the total bacteria.

Finally, a total of 103 bacterial species (Table S1) and one Miscellaneous Euryarchaeotic Group (MEG, archeae) were detected. Only a few genera were shown to include more than one species, including *Bacillus, Burkholderia, Enterobacter, Neisseria, Prevoltella, Providencia*, and *Serratia*.

Of note, sequences assigned to *W. glossinidia* showed a relative abundance higher than 80% in all except two samples (samples 20 and 35, relative abundance 51.27 and 34.55%, respectively; Figure S3). A high number of sequences assigned to the *Pseudomonas, Sphingomonas*, and Hyphomicrobiaceae were detected in sample 26, and 74 species were found in sample 32.

### Bacterial communities between infected and uninfected flies from the different geographic located HAT

The average number of bacterial species per tsetse sample was not significantly different regarding their group (Kruskal–Wallis *p-value* = 0.342), their geographical origin (Kruskal–Wallis *p-value* = 0.167), or their status (infected vs. non-infected; Kruskal–Wallis *p-value* = 0.836).

**Figure 6:**
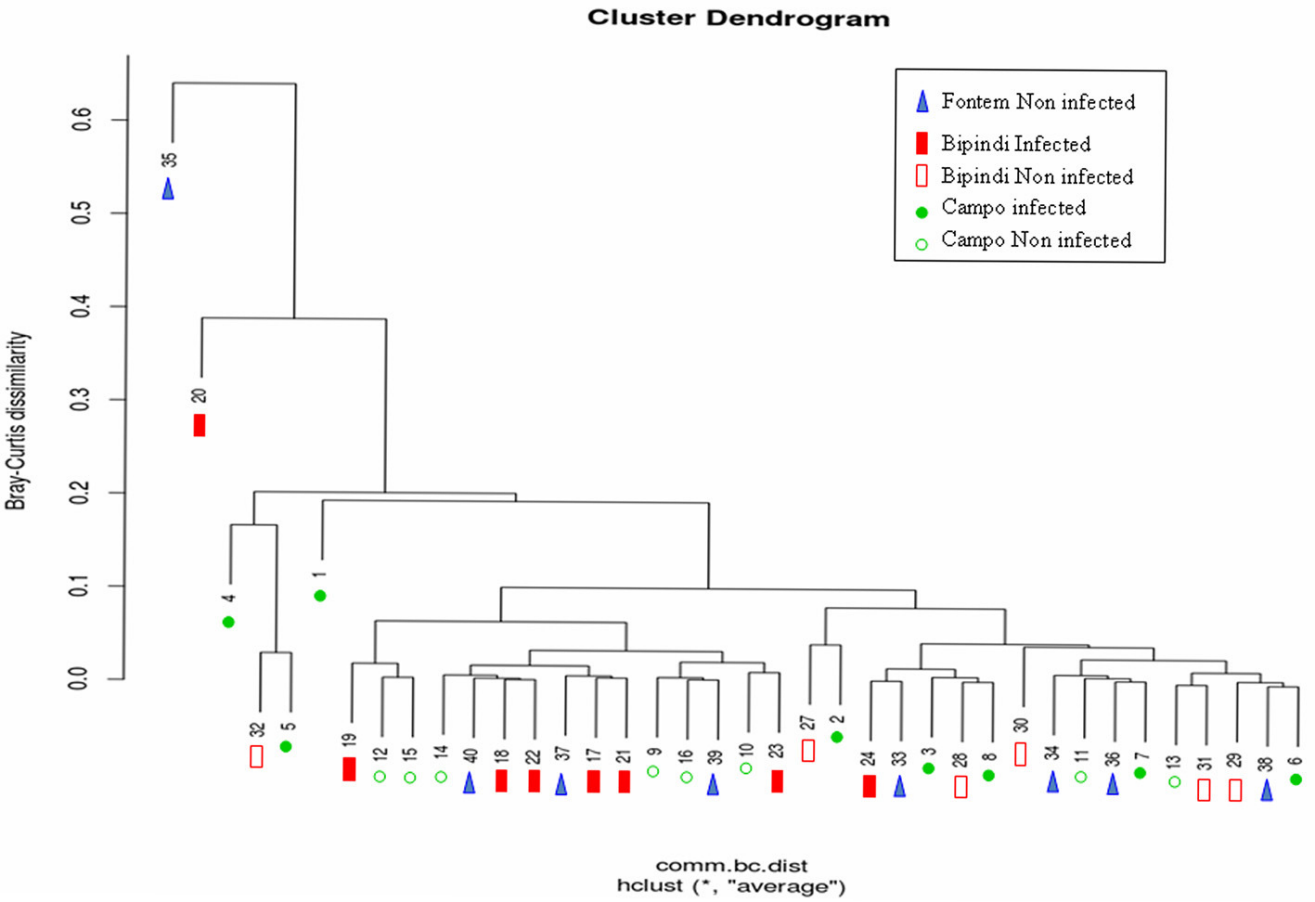
Hierarchical cluster dendrogram based on Bray-Curtis Index values, showing the relationship between different samples (represented by numbers) and foci. Sample numbering as in Figure 4.

We investigated potential relationships between the structure of the gut microbial communities of tsetse flies from Campo, Bipindi, and Fontem foci and the fly's trypanosome infection status. A hierarchical clustering using the Bray–Curtis indice did not discriminate unambiguously the different groups (Figure 6). It however showed a trend of infected flies from a given focus to be separated from non-infected flies of the same focus. One can also notice that infected flies from Bipindi are closely related to non-infected flies from Campo and vice versa. In contrast, the non-infected samples from Fontem were distributed over the different clusters. These results were reinforced by the nonmetric multidimensional scaling plots shown in Figures S4A,B.

### Novel bacteria taxa

A phylogenetic tree was inferred to further investigate the potential dissimilarities between the bacteria sequences identified in this study and the sequences currently available in public databases, thereby illustrating the taxonomic relationships between the representative OTU sequences (Figures S5A–D). The most abundant OTU present in each of the 40 samples showed sequence similarities with *Wigglesworthia* sp. We identified 60 different genotypes of *Wigglesworthia*, similar to those from *G. morsitans* or *G. brevipalpis*. Depending on the sample, there were one to four genotypes per sample. However, most samples harbored one genotype, except those from Fontem that harbored two to four different genotypes in the same sample.

**Figure 7:**
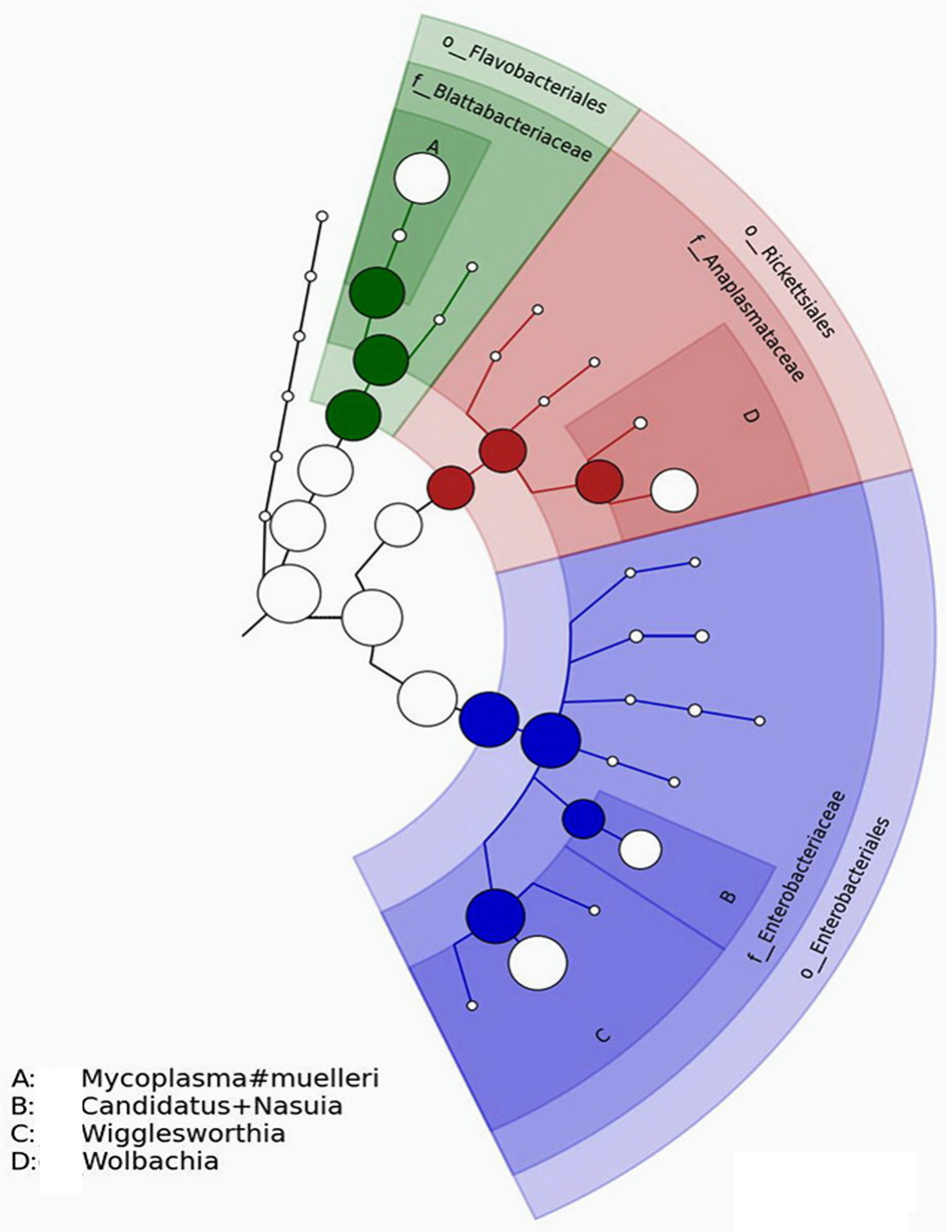
Phylogenetic diversity of bacterial *16S rRNA* sequences found in sample 20 (infected tsetse fly midguts from Bipindi).

**Figure 8:**
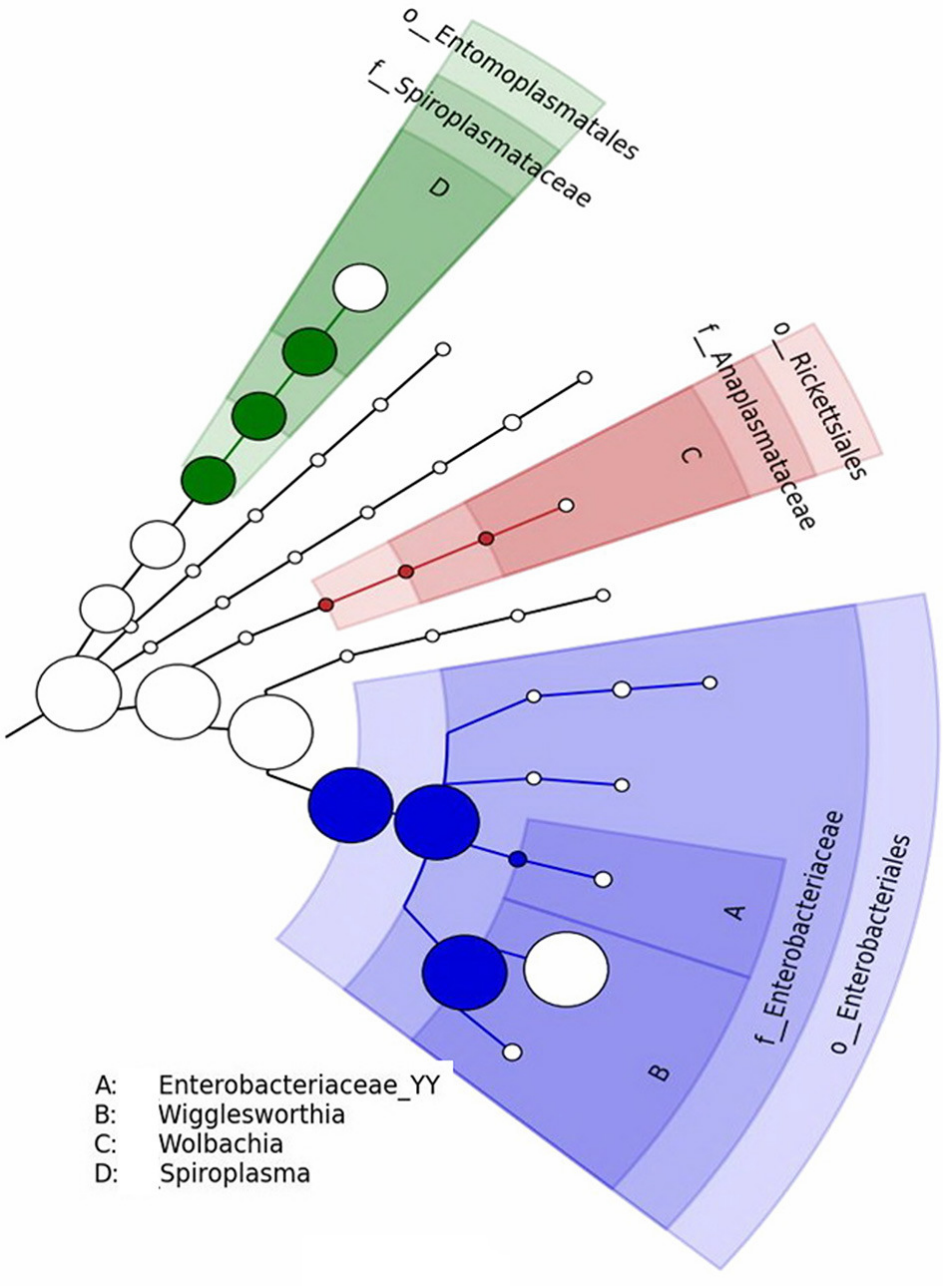
Phylogenetic diversity of bacterial *16S rRNA* sequences found in sample 30 (uninfected tsetse fly midguts from Bipindi).

Some OTU-representative sequences did not match known targets in the NCBI database (analyses performed in 2014) and had to be annotated manually. Sample 20 (Figure 7) was shown to harbor four different genotypes of several bacteria species, including *Candidatus Nasuia* sp., *Wigglesworthia* sp., and *Wolbachia* sp. Similarly, four different genotypes of *Enterobacteriaceae* sp., *Wigglesworthia* sp., *Wolbachia* sp., and *Spiroplasma* sp. were detected in sample 30 (Figure 8).

### Presence of symbionts

Special attention was paid to symbionts, due to their role in tsetse physiology and their interaction with trypanosomes. *Wigglesworthia glossinidia* was once again characterized by its high density in most samples and groups; this was assessed by the high percentages (more than 80% in each sample), except in two samples from groups 4 and 5 (Bipindi and Fontem, non-infected flies). *Wolbachia* sp. sequences were also present in all groups, but in smaller amounts. Nevertheless, a very high number of *Wolbachia* sequences were observed in sample 20 (11.08%). In addition, we detected, at low relative abundance, the presence of the symbiont *S. glossinidius* in four samples distributed over all groups, except Bipindi-uninfected (group 4).

## Discussion

The main objective of this study was to characterize the bacterial communities inhabiting the midgut of field-collected tsetse flies from sleeping sickness foci in Cameroon using deep sequencing. Trypanosome-infected and non-infected flies were sampled in three sleeping sickness foci in Cameroon (Bipindi, Campo, and Fontem) that differ in their natural environment, thus offering the possibility to investigate the potential association between specific bacterial communities composition and the environment and/or the ability of the flies to be infected by trypanosomes. In this study the targeted trypanosome species was *T. congolense* (causing AAT) since, as shown in Figure 3, its prevalence was large enough in both Campo and Bipindi sampled flies. In contrast, *T. brucei* (*sensu lato*, Tb *sl* hereafter) had a much lower prevalence. Indeed, only 6 Tb *sl* infected flies were recorded in the sampled sites which, furthermore, included the two sub-species, *T. brucei brucei* and *T. brucei gambiense*—the former causing AAT and the second being responsible for the chronic form of HAT.

The Campo sampling campaign was performed in 2008, while the ones regarding Bipindi and Fontem were performed in 2013. A reduced but significant gene flow, due to fly migration, was previously reported between Campo and Bipindi (Farikou et al., 2011b), raising the question whether the 5 years delay between the sampling periods could affect the fly infection status and the microbiota profiles. One may consider that in the case of an important and/or continuous migration flow for years, differences in fly population diversity between the two foci would have been reduced or even suppressed. This was clearly not the case, and was never recorded (Farikou et al., 2010a,b). Although, the possibility that the 5 years delay could marginally affect the comparison between the 3 foci cannot be totally excluded, it could not affect the intra-foci comparison between trypanosome infected and non-infected flies.

### Diversity of the bacterial communities and its origin

We detected a very large, unexpected number of different bacterial taxa (103 species), among which 26 were found in more than one sample. The number of different species identified in the present study was clearly much higher than in previous reports. For instance, three species were identified in *G. p. palpalis* in Angola (Geiger et al., 2009); one, three and eight species were, respectively, found in *G. nigrofusca, G. pallicera*, and *G. p. palpalis* in Cameroon (Geiger et al., 2011b); one species was identified in *G. p. gambiensis*; and 24 species were found in *G. f. fuscipes* in Kenya (Lindh and Lehane, 2011). With the exception of two bacterial species, all species detected in these previous studies were identified using culture-dependent methods. Many bacteria species are difficult to culture and the density of some species may be too low for proper isolation. This could explain the low number of bacteria identified in other studies as compared to the high number of species we characterized in the gut of tsetse flies using molecular approach.

A more recent study (Aksoy et al., 2014) identified a high number of bacteria species in the gut microbiota of several tsetse fly species collected in Uganda using molecular approaches, although the bacteriome of Uganda flies was less diverse than the one of flies from Cameroon.

Compared to the bacterial communities of flies from Uganda, flies from Cameroon revealed both similarities (namely Enterobacteriaceae and an unexpected high abundance of *Wigglesworthia*) and differences (bacteria belonging to the genus *Delftia, Stenotrophomonas, Thermoanaerobacterium, Proteus*, and *Ochrobactrum*, among others, were not found in flies from Cameroon). These differences may be due to differences in the respective investigated fly species, *G. f. fuscipes, G. m. morsitans, G pallidipes* vs. *G. p. palpalis*, and/or the respective environmental factors and the geographic locations of the HAT/AAT foci in Uganda vs. Cameroon.

Aside from the question of what effect these gut bacteria may have on tsetse fly physiology and/or on their ability to become trypanosomes infected (i.e., their vector competence), there is also the question of how tsetse flies can acquire such a large diversity of bacterial species. In their report, Aksoy et al. (2014) mentioned the possibility that some of the low abundant bacteria “may have arisen from contamination from environmental sources, such as during field-based dissection processes,….” Although, the occurrence of contamination can never be totally excluded, our data do not support this comment since it would be expected, in such case, that several bacteria species from the environment would be detected, at low abundance, in all or most of the samples, inasmuch the 40 fly were processed at the same time. As shown in Table S1, this is not the case; most of the low abundant bacteria (74 out of 103) are present in only one sample and, in contrast, one of the samples (number 32) was shown to harbor 59 species. Nevertheless, further independent studies should be undertaken to confirm the finding. An alternative explanation could account for the complexity of the microbiota as it was recently reported, that is, tsetse flies are not strictly hematophagous; they can occasionally feed on nectar from a broad range of plants growing within their different ecological environments, and, thus, may ingest bacteria colonizing this nutriment (Colman et al., 2012; Solano et al., 2015). Such effect of diet differences and their links to environmental conditions, on bacteriome community structure of insects has been documented by Colman et al. (2012). Finally, as reported by Poinar et al. (1979), adult flies may be contaminated with bacteria present on the epidermis of humans or animals they bite during blood meal. This could lead to a large diversity of the fly bacteriome since tsetse flies are known to ingest their blood meals from a variety of vertebrates (Moloo et al., 1988; Simo et al., 2008; Farikou et al., 2010b). Thus, despite the viviparous reproduction of tsetse flies (in contrast to other dipterans) that prevent fly contamination at the larval stage (except for the symbionts that are vertically transmitted from the mother to the larva *via* milk gland secretions), the modes of acquiring “exogenous” bacteria by tsetse flies seems to be more diverse than previously believed and could explain the recorded large microbiota diversity herein.

One however has to keep in mind that in addition to true tsetse gut inhabitants, it is likely that some—or many—of the 143 OTUs are solely opportunistic, simply in transit or possibly present only as DNA fragments. Since dead bacteria provide the same DNA signature as do living bacteria, further investigations should aim to isolate bacteria of interest; only such approach will provide best evidence of their presence as a living organism in the tsetse gut.

### Bacterial species richness of the samples (alpha diversity)

Although, the analyzed midgut bacteriome of adult tsetse flies included 103 species belonging to 13 phyla, only 4 of them were dominant (Proteobacteria, Bacteroidetes, Actinobacteria, and Firmicutes), showing some similarities with gut microbial communities from other invertebrates such as cattle tick (Andreotti et al., 2011), several species of *Anopheles* (Rani et al., 2009; Boissière et al., 2012) and of *Aedes* (Zouache et al., 2011; Terenius et al., 2012); Acridid (Dillon et al., 2008); Asian longhorned beetle (Geib et al., 2009); *Lutzomyia longipalpis* (Gouveia et al., 2008); honey and bumble bees (Martinson et al., 2011). However, there is a major difference between the bacteriome of all these invertebrates and those of tsetse flies, namely the presence, in the latter, of *W. glossinidia* in all samples. *W. glossinidia*'s presence is not surprising as it is the tsetse fly's obligate symbiont; its abundance in the different samples was however remarkable as the mean relative abundance over the 40 samples was as high as 95.3% (even higher than 99% in all but five samples—see below), while that of *Enterobacter homaechei*, also present in all samples, was only 1.8%. Such an “overabundance” of *Wigglesworthia* was previously reported by Aksoy et al. (2014) who estimate that it may disrupt proper identification of low abundant bacteria thus leading to an underestimation of the alpha diversity (species richness). In order to decrease the abundance of this bacterium—and thus overcoming the difficulty related to its overabundance—the group of Aksoy et al. (2014) excised part of the bacteriome organ harboring *Wigglesworthia* and was able to discover some bacteria previously non-identified when analyzing the whole bacteriome. In our study, five samples showed smaller *Wigglesworthia* relative abundance than the 35 others; these are [sample number and (relative *Wigglesworthia* abundance)] sample 2 (88.83%), 4 (80.52%), 20 (51.27%), 32 (86.96%), and 35 (34.55%) while the numbers of hosted bacteria were, respectively, 16, 9, 8, 73, 10 (*Wigglesworthia* excluded). Thus, concerning our study, it seems that there was no relationship between the *Wigglesworthia* relative abundance and the species richness of the samples [for example, sample 32 hosted the largest number of bacteria although the relative abundance of *Wigglesworthia* was rather high (86.96%)]. Thus, as suggested by Aksoy et al. (2014), further investigations on the tsetse fly bacteriome should be performed using genomic/complementary functional approaches.

### Microbiome composition and the possible role of identified bacteria

Most of the sequence tags corresponded to few taxa. Only 16 species displayed a maximum relative abundance per sample higher than 1%, whereas 78 species had a maximum relative abundance lower than 0.2%. Nevertheless, some members of the latter group of bacteria are potentially of great interest.

#### The symbionts

This group of bacteria includes in particular three species *W. glossinidia, S. glossinidius*, and *Wolbacchia*. Besides its prominent role as an obligate symbiont of the tsetse fly, *Wigglesworthia* differs from the other bacteria by the fact that 60 genotypes of this species have been identified, whereas most other bacteria were represented by only one genotype, or at least by a limited number of genotypes. In samples from Uganda, differences were also detected in the sequences of the *Wigglesworthia* V4 region (Aksoy et al., 2014) that confirmed the existence of several genotypes, although the authors reported small amounts of *Wigglesworthia* carryover between the different tsetse species sampled; thus they do not exclude completely the occurrence of a cross contamination. Nevertheless, the occurrence of multiple genotypes of a given species is not a surprise and may not be considered as an artifact generated along the technical process. For example, we have previously identified number of *S. glossinidius* genotypes when using other approaches than amplicon sequencing (Illumina MiSeq) such as AFLP (Geiger et al., 2005, 2007) or microsatellite (Farikou et al., 2011a,b) genotyping on either insectary-reared or field sampled tsetse flies. This finding could be of great interest, as until now only the density of *Wigglesworthia*, not its potential genetic diversity, was considered to be involved in the modulation of tsetse fly infection by trypanosomes (Wang et al., 2009). In fact, the genetic diversity within populations of *W. glossinidia* that infect tsetse flies could play a prominent role, as previously demonstrated for the facultative symbiont *S. glossinidius* (Geiger et al., 2007). However, investigating a possible association between trypanosome infection and specific *W. glossinidia* genotypes (60 have been identified) will require a much larger sampling size.

Attention was paid to *Sodalis* that was previously shown to be involved in modulating the tsetse fly's ability to acquire trypanosomes (Farikou et al., 2010a; Hamidou Soumana et al., 2014). In the present study this symbiont was identified in 10% of the samples, a finding markedly different from the 30% average prevalence we found previously in nearly the same sampling sites (Farikou et al., 2010a), and from that recorded for example by Aksoy et al. (2014) who suggest the variations could be a matter of sensitivity of the analytical approach. However, this could be only part of an explanation since we used in our study similar analytical approaches and instruments (including the Illumina MiSeq platform). Overall, these data confirm the existence of a very high symbiont variability in natural populations of flies, as previously reported by Farikou et al. (2010a). These variations could be linked to a multitude of factors including differences in tsetse individual flying times, field environment modifications or, as suggested by Aksoy et al. (2014) the competition between *Sodalis* and other intestinal bacteria which diversity and respective abundance may vary.

*Wolbachia* sp. was previously characterized in field populations of *G. morsitans morsitans, G. morsitans centralis, G. austeni* flies, *G. brevipalpis, G. pallidipes*, and *G. p. gambiensis* (Doudoumis et al., 2012). Interestingly, *Wolbachia* has not been characterized in *G. fuscipes fuscipes* or *G. tachinoides* and, until now in *G. p. palpalis*. The bacterium was detected in 25 out of 40 flies (62.5%) and was distributed over the 5 groups (thus across the three sampling sites, and the fly trypanosome infection status): 5, 4, 7, 5, and 4 *Wolbachia* positive flies in groups 1–5, respectively. At this stage of the investigation we cannot suggest any explanation for its presence; we can only report its relatively high prevalence. The fact that until now *Wolbacchia* was never characterized in *G. palpalis palpalis* does not mean such infection does not occur in other fly individuals; further studies should focus on surveys of larger *G. p. palpalis* populations from the studied HAT foci, or on populations from HAT foci located elsewhere than in Cameroon (for example flies from HAT foci located in RDC—République Démocratique du Congo—or in Angola). In a recent report, Ji et al. (2015) highlight the extreme variability (from 1.54 to 66.67%) of the prevalence of *Wolbacchia* across 25 *Bemisia tabaci* populations from China. The frequency of *Bemisia* infection was shown to be affected by a large panel of factors (putative *B. tabaci* species, geographic location, sex of the host …). Finally, a cystoplasmic incompatibility occurs when a *Wolbacchia* negative (W−) female fly mates with a *Wolbacchia* positive (W+) male, and the embryos degenerate (Alam et al., 2011). In contrast, when a (W+) female mates with a (W+) or a (W−) male, the offspring is not only fertile but also more numerous than in the case of a (W−) female with (W−) male mating. As *Wolbacchia* is transmitted by the female fly to its offspring, the presence of *Wolbacchia* in a female gives them a reproductive advantage. Could this explain the high prevalence of *Wolbacchia* in the studied Gpp populations?

In contrast to *Anopheles gambiae* whose susceptibility to *Plasmodium* infection increased with the presence of *Wolbachia* under natural conditions (Zélé et al., 2014); the presence of this symbiont was not found to be associated with the infection status of the tsetse flies.

We also identified in tsetse fly midguts the presence of bacteria that are known to be symbionts when infecting other organisms. This is the case of *Candidatus Sulcia muelleri* (Flavobacteriaceae), which displayed an ancient symbiotic relationship with the hemipteran *Auchenorrhyncha* (Moran et al., 2005). The identified bacterium *Candidatus Zinderia* is also a symbiont of the tick *Haemaphysalis longicornis* (McCutcheon and Moran, 2010). Finally, we observed the cohabitation of four symbionts in some of our analyzed samples, although it is unknown if they develop a synergistic effect on the fly's physiology. Given the current state of our investigation, large-scale analyses (including attempts to isolate the two *Candidatus* species) deserve to be undertaken, as until now only three symbionts have been described in tsetse flies.

#### Other bacteria of interest

*S. marcescens* and *Serratia odorifera* (Enterobacteriaceae) were present in more than 50% of the samples. Both species are considered to be opportunistic pathogens for humans (Chmel, 1988; Hejazi and Falkiner, 1997; Grimont and Grimont, 2015). *Serratia marcescens* is also responsible for the increased mortality in tsetse flies (Poinar et al., 1979) such as *Glossina pallidipes* (Gonzalez-Ceron et al., 2003); it secretes trypanolytic compounds and reduces the establishment of *T. cruzi* in the midgut of its vector, *Rhodnius prolixus* (Triatominae), vector of *T. cruzi* causing the Chagas disease; Azambuja et al., 2004). One previous work reported the presence of the novel species *S. glossinae* in the gut of insectary-reared *G. palpalis gambiensis* flies (Geiger et al., 2010), although this species has never been identified in field-collected *Glossina* flies. Surprisingly, the same situation was observed for mosquitoes where the bacterium *Elizabethkingia* was found only in insectary-reared *Anopheles* sp. (Boissière et al., 2012; Gimonneau et al., 2014). However, this phenomenon cannot be currently explained. The presence of *Serratia* in the guts of *G. f. fuscipes* flies from western Kenya has also been reported (Lindh and Lehane, 2011). In *Anopheles gambiae* mosquito midguts, multiple strains of *Serratia* have been identified, some of which are able to reduce mosquito infections by *Plasmodium* (Bando et al., 2013). *Serratia* has also been identified as a newly acquired symbiotic partner in aphids (Burke and Moran, 2011). Currently, we did not evidence a clear association between the presence of some bacterial and trypanosome infection. However, as suggested previously by Aksoy et al. (2014), understanding the association of *Serratia* with tsetse flies and its functional role in tsetse physiology could provide critical knowledge about trypanosome transmission dynamics in tsetse flies. Similar investigations could be performed with other bacteria.

In addition to *Serratia*, other identified bacteria have the ability to produce antiparasitic compounds, including members of *Acinetobacter*. This genus was previously characterized in the gut of tsetse flies from Cameroon (Geiger et al., 2009). The presence of *Acinetobacter* (*guillouiae* and spp.) was observed in 6 samples in the present study.

### Association between the composition of the intestinal communities and the fly infection status and/or the environmental differences

The characterization of the composition of the intestinal bacterial communities was an important objective of the work. However, the statistical analyses of the identified bacteria in this study did not reveal any significant differences in the sample bacterial species richness between the different groups (Campo and Bipindi infected and uninfected flies as well as Fontem uninfected flies), or the status of the flies (infected vs. uninfected). A hierarchical clustering neither discriminate properly the different groups, nor the infected flies from the non-infected flies of the same focus. At the level of individual bacteria, some of them seem to display an association with a given group or a sampling site. However, most often they concern species that are poorly represented. The possibly that environmental differences between the three sampling sites (especially the climate, wildlife, and flora) were too weak to induce significant differences in the flies' microbiome composition may be considered. These data suggest future fly microbiome research should aim for greater numbers of flies and target more ecologically contrasted sites, in order to identify bacteria involved in (or at least associated with) the fly vector competence.

In conclusion the meta-barcoding analysis that we performed on DNA extracted from *G. p. palpalis* collected from three different HAT foci in Cameroon resulted in the detection of 143 OTUs belonging to 83 genera and 13 phyla. Part of these bacteria could be identified at the species level (103 annotated OTUs). However, some species were not identifiable since the corresponding sequences did not match any available sequence libraries; they may correspond to novel species, and need to undergo further identification process. The presence of such a variety of bacteria was unexpected and raises many questions regarding how they are acquired by tsetse flies, and the role they may play regarding the fly's physiology and vector competence. In addition, it would be of interest, in a following investigation campaign, to enlarge the sampling scheme and include teneral flies as a supplementary control besides non-infected non-teneral flies. The former are expected to host a more limited microbiota and to have a less developed immunity compared to the latter. On the other hand, it should be noted that, when teneral flies undergo artificial infection process, about 70% do not develop trypanosome infection (Geiger et al., 2007). This suggests that within a collection of teneral flies most of them, even considered as “naïve,” are physiologically and genetically “programmed” to be/become refractory to trypanosome infection.

## Author contributions

Conceived and designed the experiments: FJ, AG. Performed the experiments: FJ, TM, GN, FN, AG. Analyzed the data: FJ, RC, AG. Contributed reagents/materials/analysis tools: AG. Wrote the paper: GG, FN, LA, JR, AG.

### Conflict of interest statement

The authors declare that the research was conducted in the absence of any commercial or financial relationships that could be construed as a potential conflict of interest.

## Supplementary material

Table S1 Classification of the total bacteria detected in the 40 tsetse samples from the Campo, Bipindi, and Fontem foci in Cameroon. The table provides, for each sample, the relative abundance (in %) of each bacterium present in this sample. Several of the detected bacteria could not be identified at the species level. The suffix “X or Y” refers to “unclassified” at that rank.

Figure S1 Rarefaction analysis on the studied samples (Renyi diversity plots); **(A)** Samples 1–24 and 27–40; **(B)** Samples 25 and 26 that were processed apart.

Figure S2 Heat map analysis of the distribution and abundance of the bacterial genera in midgut samples. The samples did not cluster together, indicating that the bacterial community among them is highly variable. Sample numbering as in Figure 4.

Figure S3 Heat map analysis of the repartition and abundance of bacterial species in midgut samples. The samples did not cluster together, indicating that the bacterial community among them is highly variable. Sample numbering as in Figure 4.

Figure S4 **(A)** NMDS plot comparing bacterial OTUs in tsetse fly midguts of different foci (Bipindi, Campo, Fontem) using Bray-Curtis dissimilarity indice. **(B)** NMDS plot comparing bacterial OTUs of infected vs. non infected tsetse fly midguts using Bray-Curtis dissimilarity indice.

Figure S5 Phylogenetic diversity of bacterial *16S rRNA* sequences in tsetse fly midguts. NJ tree clustering of OTU representatives based on homology with known sequences is shown. The width of the branch is proportional to the number of samples exhibiting a given OTU. Colors correspond to different phyla. **(A–D)** Expended scale of four regions from this figure in order to make it easier to read (the corresponding regions are indicated in this figure).
