# Supplementary figures and images for: Intestinal Bacterial Communities of Trypanosome-Infected and Uninfected Glossina palpalis palpalis from Three Human African Trypanomiasis Foci in Cameroon
Source: Front Microbiol. 2017 Aug 3;8:1464. doi: 10.3389/fmicb.2017.01464 (PMC5541443; doi:10.3389/fmicb.2017.01464)

Figure S1  
Renyi diversity plots

Fig. S1 a  
Samples 1 to 24 & 27 to 40

Fig. S1 b  
Samples 25 & 26

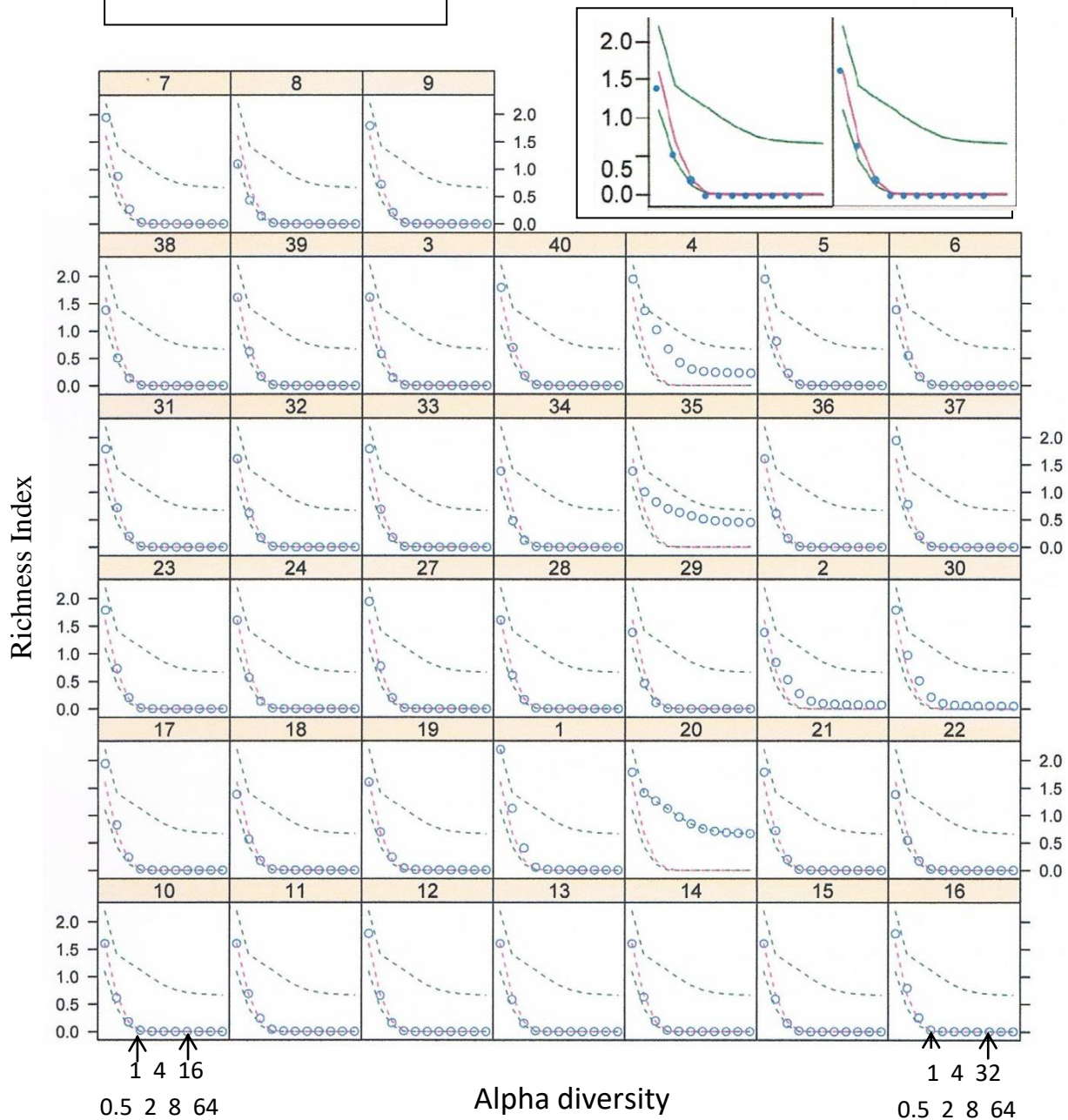

Supplement: Figure S1 — Rarefaction analysis on the studied samples (Renyi diversity plots); (A) Samples 1–24 and 27–40; (B) Samples 25 and 26 that were processed apart. [file Image1.PDF]

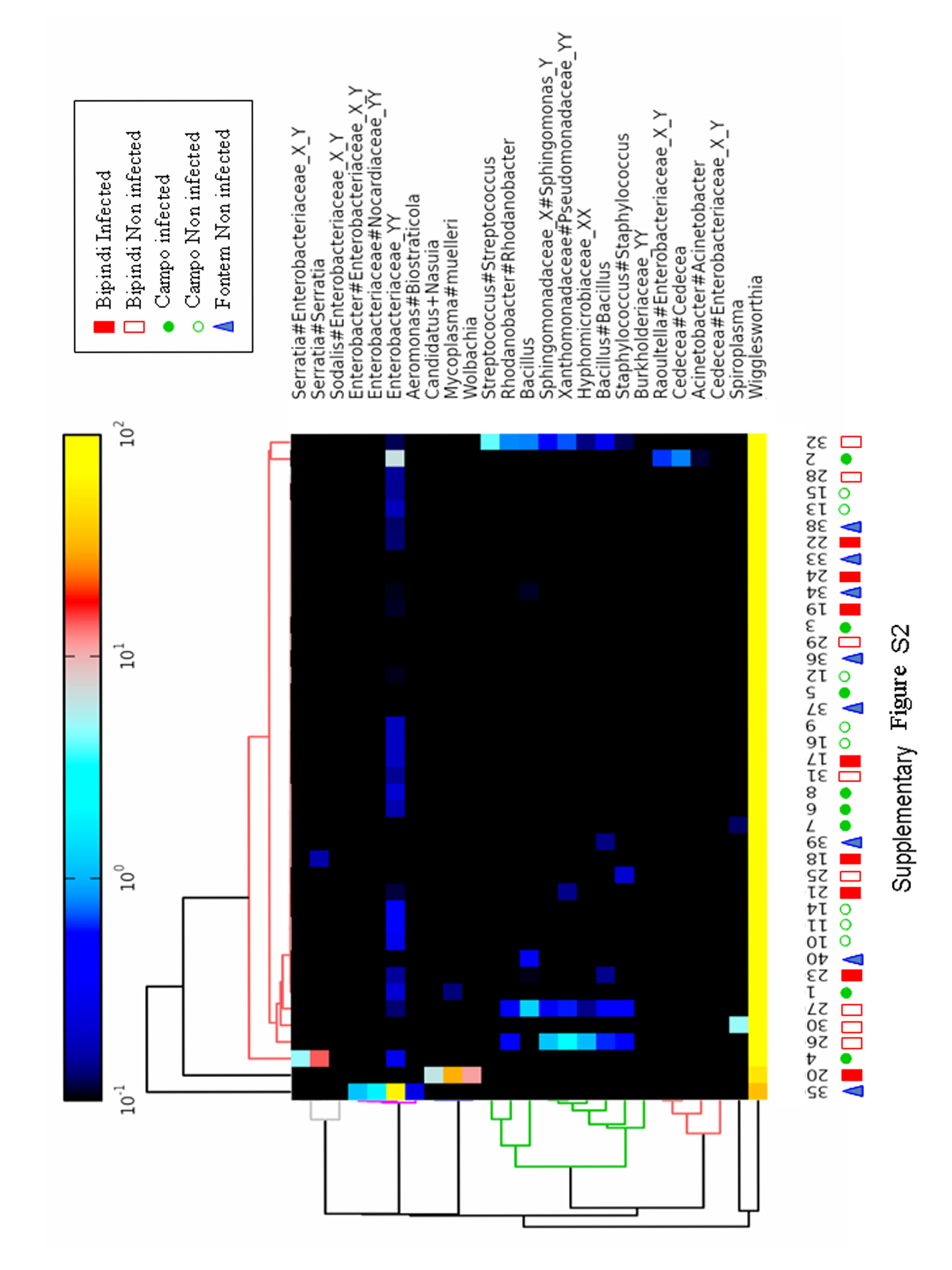

Supplement: Figure S2 — Heat map analysis of the distribution and abundance of the bacterial genera in midgut samples. The samples did not cluster together, indicating that the bacterial community among them is highly variable. Sample numbering as in Figure 4. [file Image2.TIF]

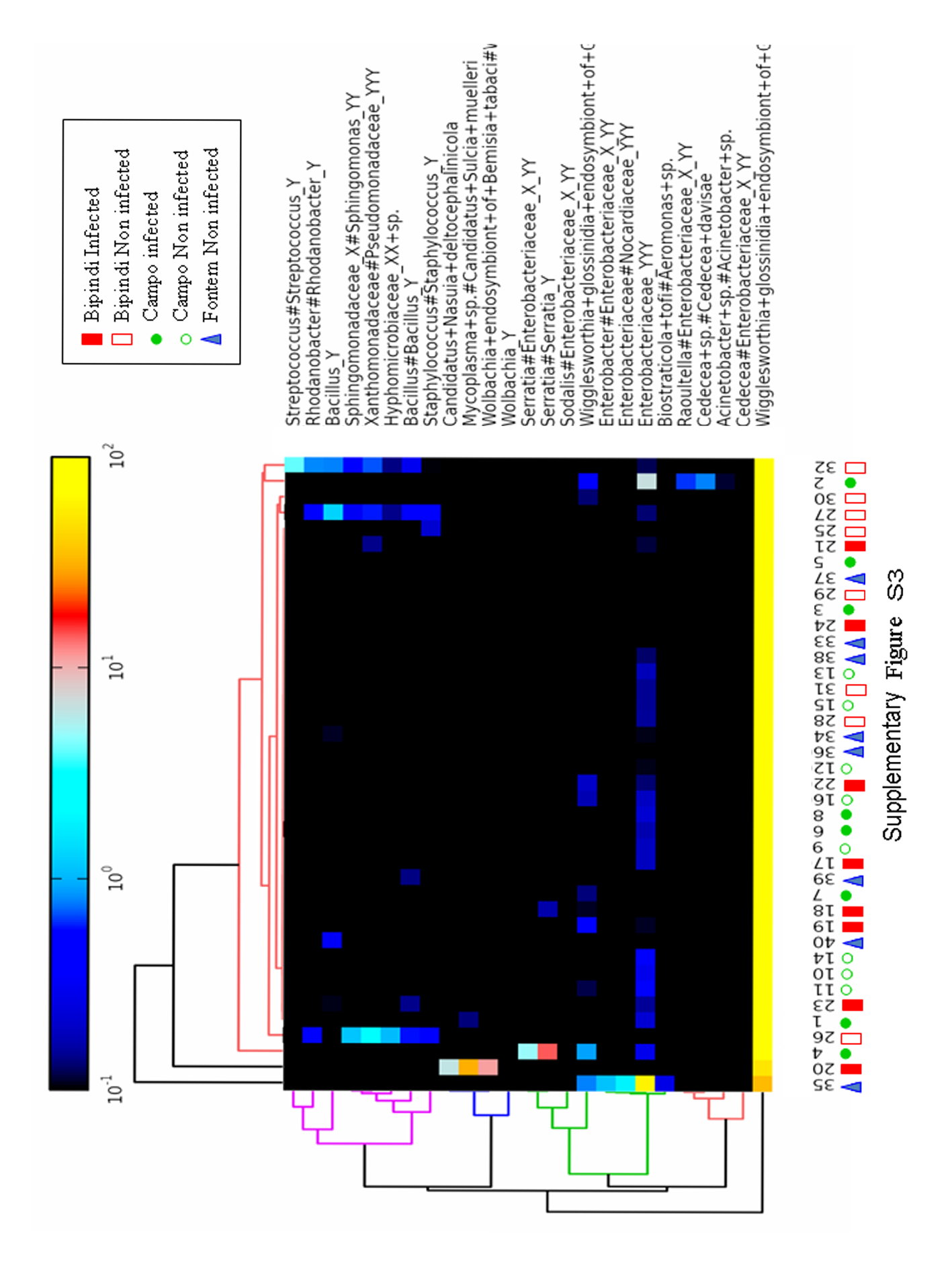

Supplement: Figure S3 — Heat map analysis of the repartition and abundance of bacterial species in midgut samples. The samples did not cluster together, indicating that the bacterial community among them is highly variable. Sample numbering as in Figure 4. [file Image3.TIF]

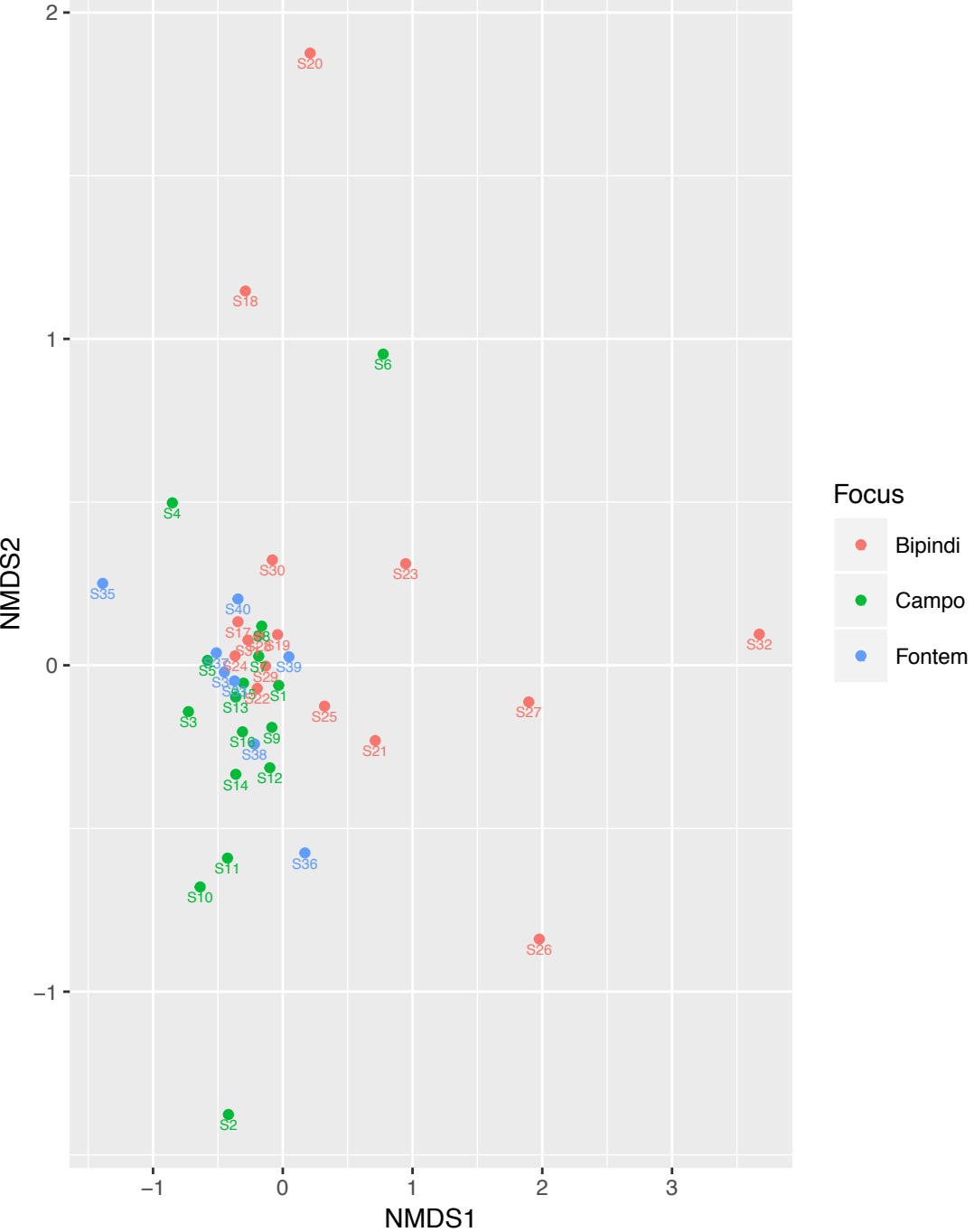

Supplementary Figure 4A

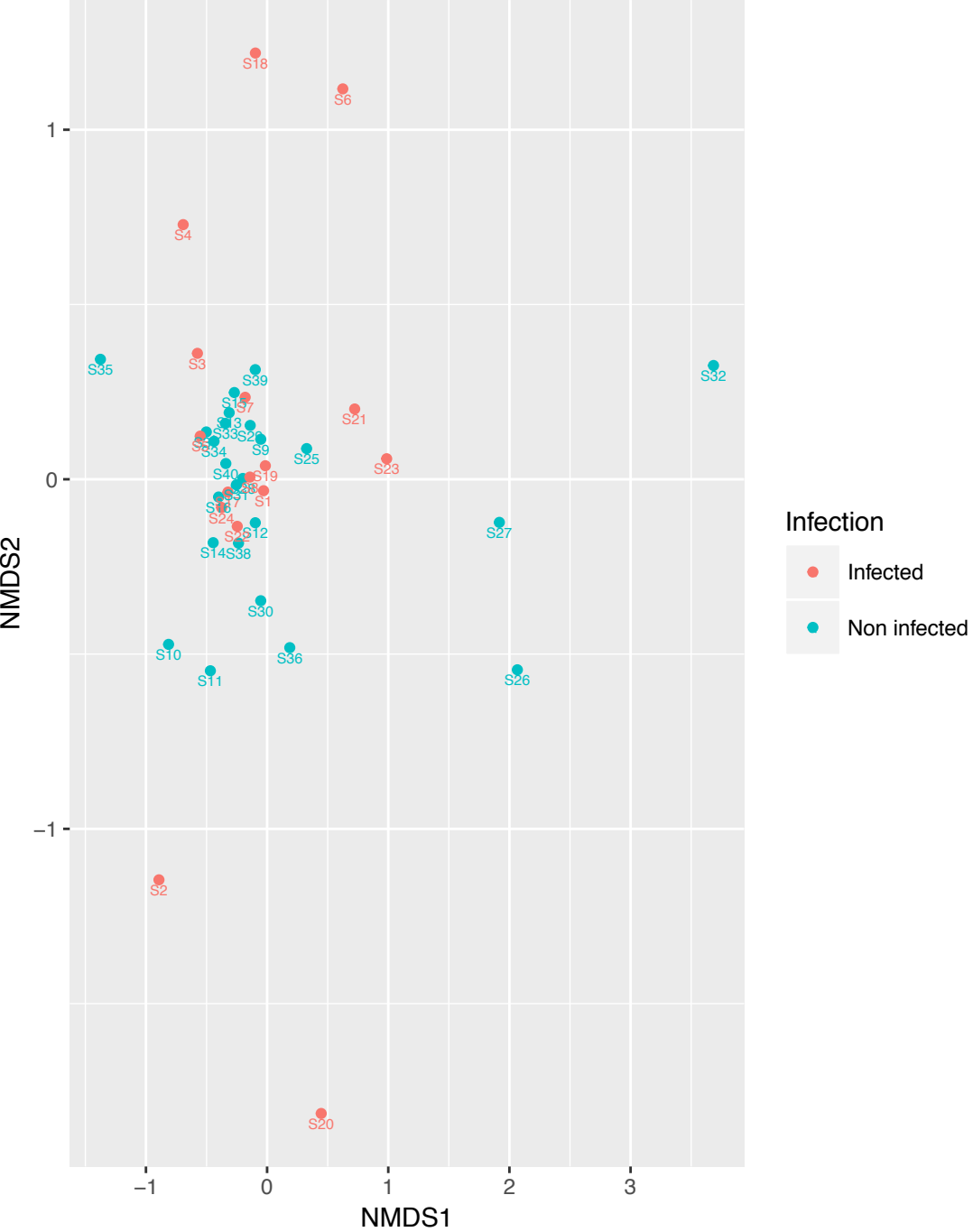

Supplementary Figure 4B

Supplement: Figure S4 — (A) NMDS plot comparing bacterial OTUs in tsetse fly midguts of different foci (Bipindi, Campo, Fontem) using Bray-Curtis dissimilarity indice. (B) NMDS plot comparing bacterial OTUs of infected vs. non infected tsetse fly midguts using Bray-Curtis dissimilarity indice. [file Image4.pdf]
