# Supplementary material for: Intestinal Bacterial Communities of Trypanosome-Infected and Uninfected Glossina palpalis palpalis from Three Human African Trypanomiasis Foci in Cameroon
Source: Front Microbiol. 2017 Aug 3;8:1464. doi: 10.3389/fmicb.2017.01464 (PMC5541443; doi:10.3389/fmicb.2017.01464)
Supplement: Figure S5 — Phylogenetic diversity of bacterial 16S rRNA sequences in tsetse fly midguts. NJ tree clustering of OTU representatives based on homology with known sequences is shown. The width of the branch is proportional to the number of samples exhibiting a given OTU. Colors correspond to different phyla. (A–D) Expended scale of four regions from this figure in order to make it easier to read (the corresponding regions are indicated in this figure). [file Image5.pdf]

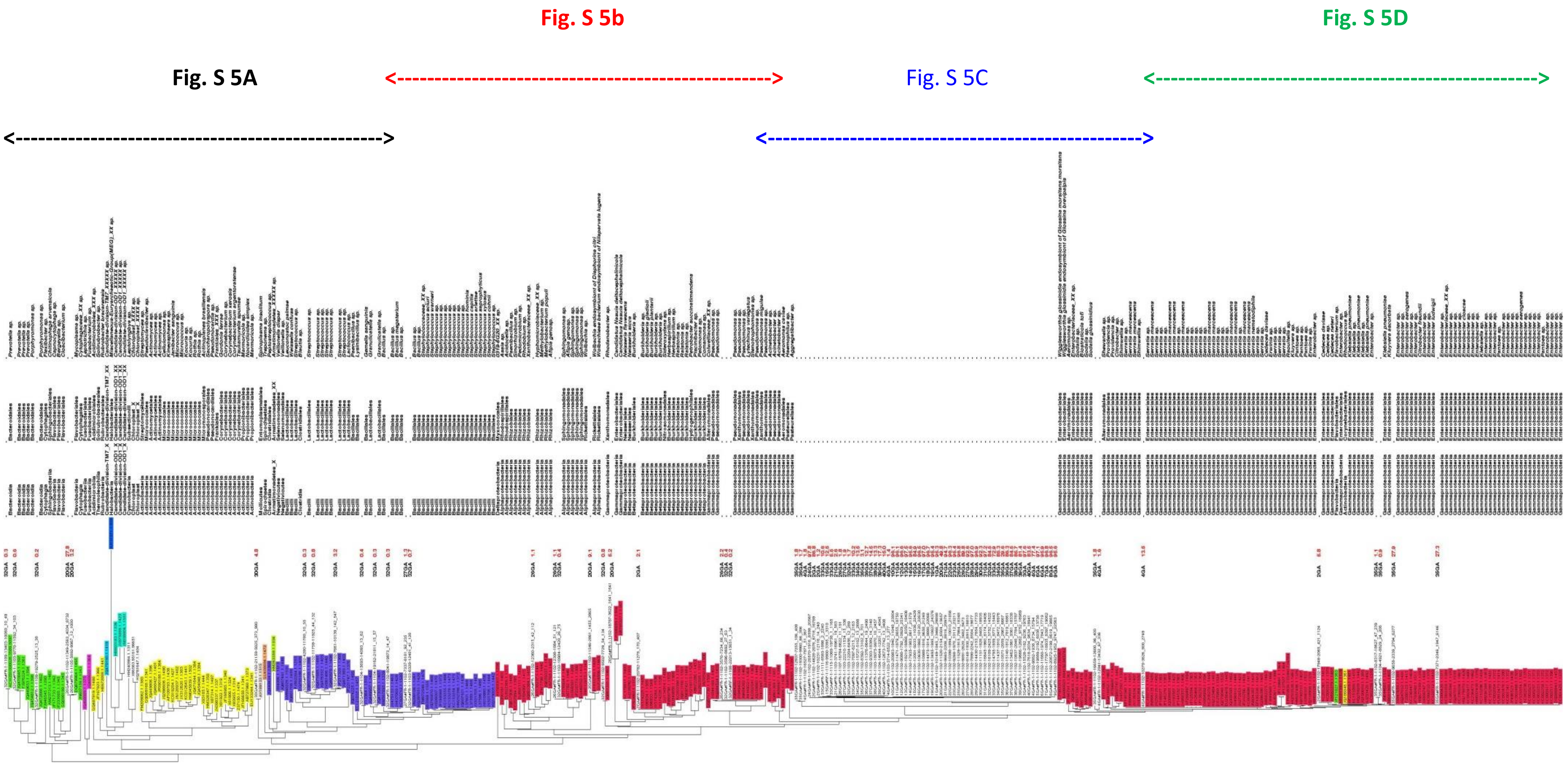


**Supplementary Figure S5**

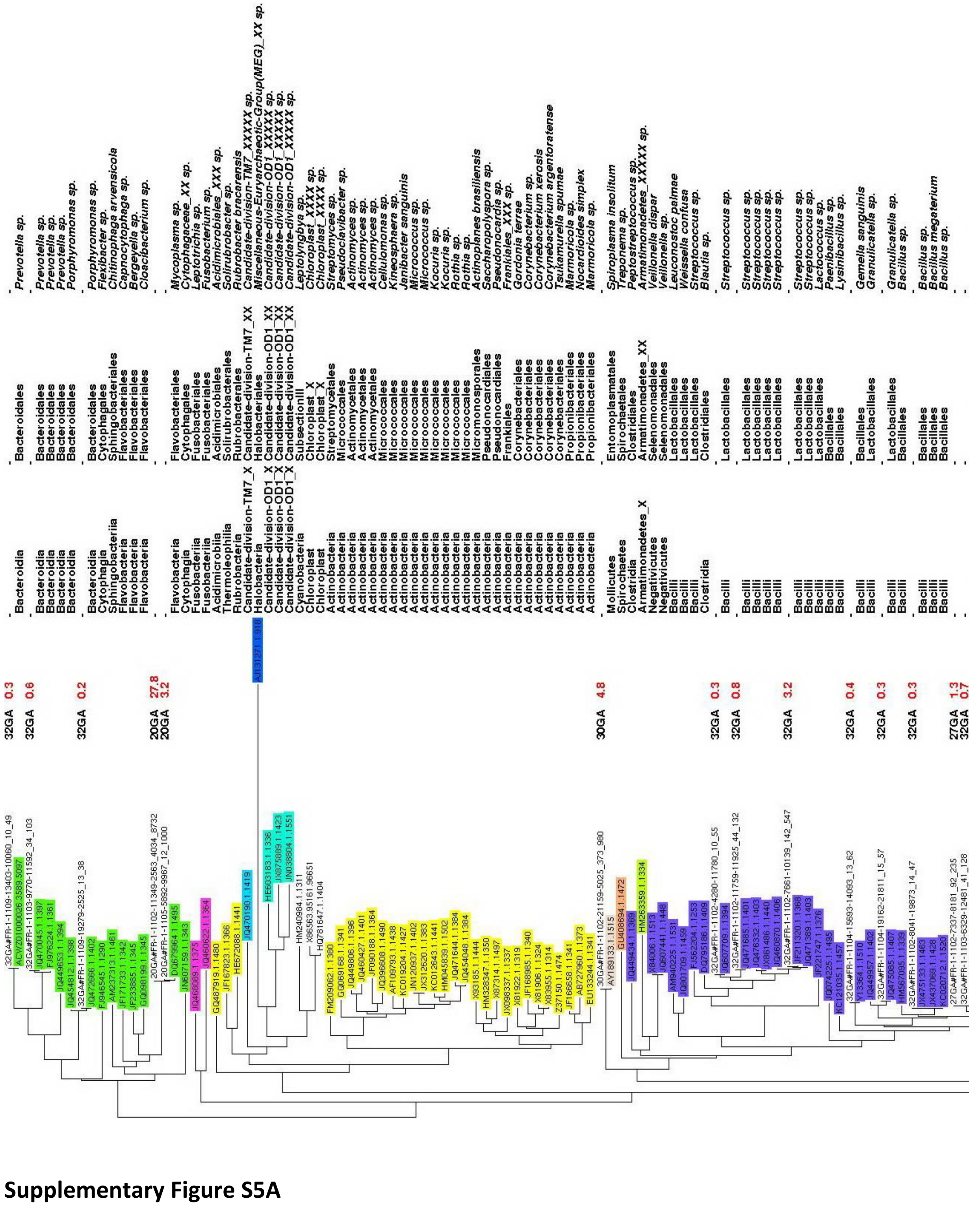

**Supplementary Figure S5A**

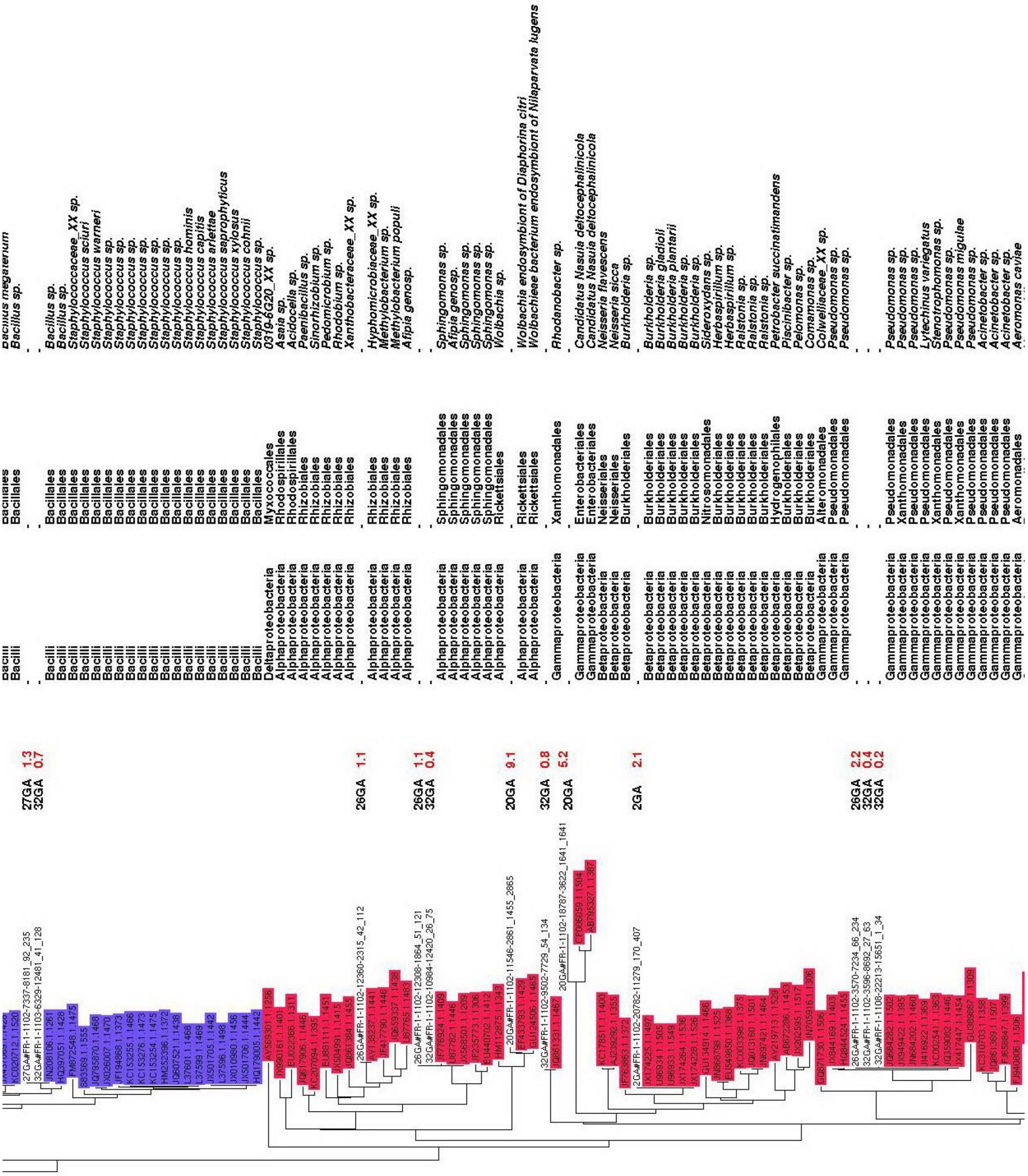

**Supplementary Figure S5B**

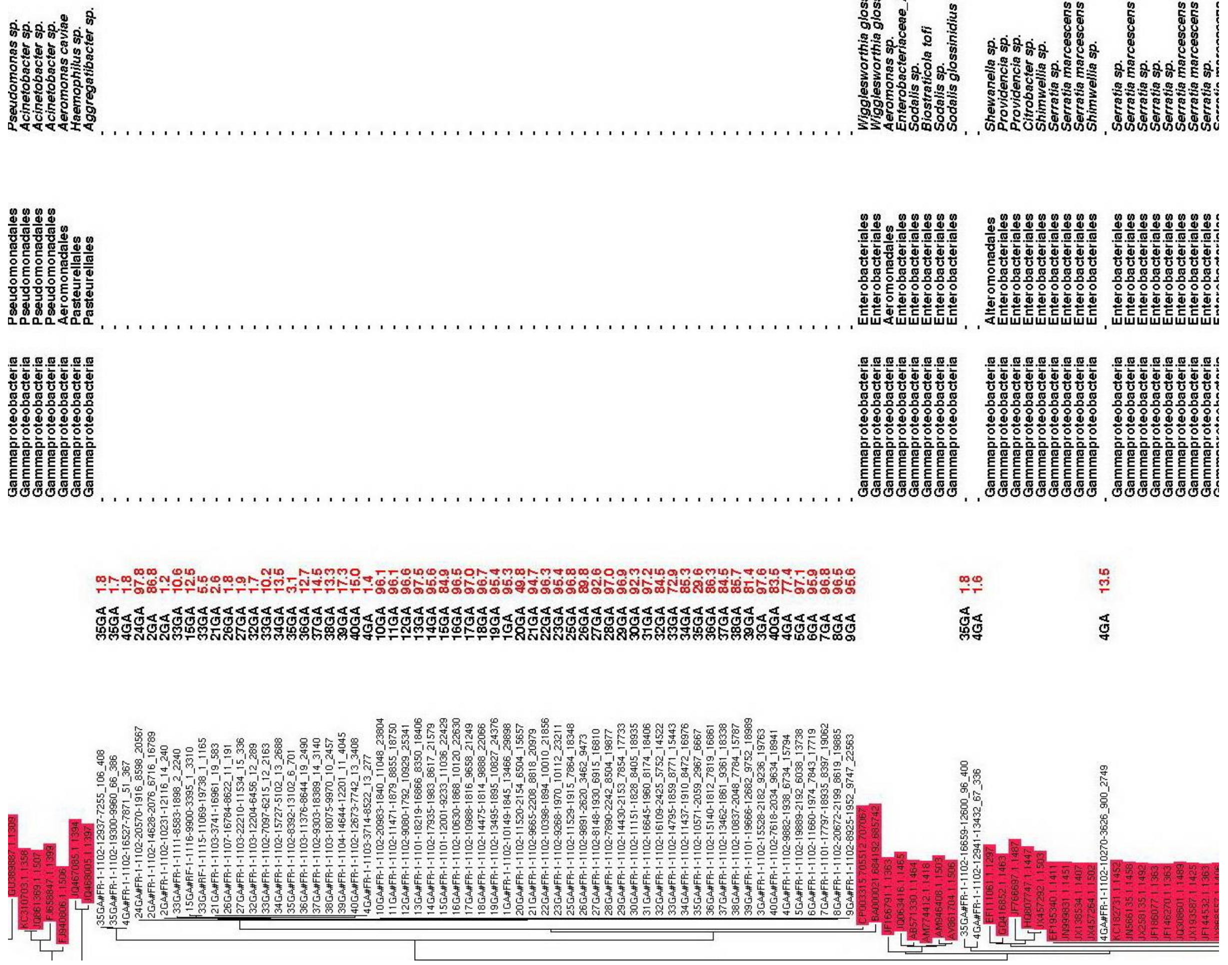

**Supplementary Figure S5C**

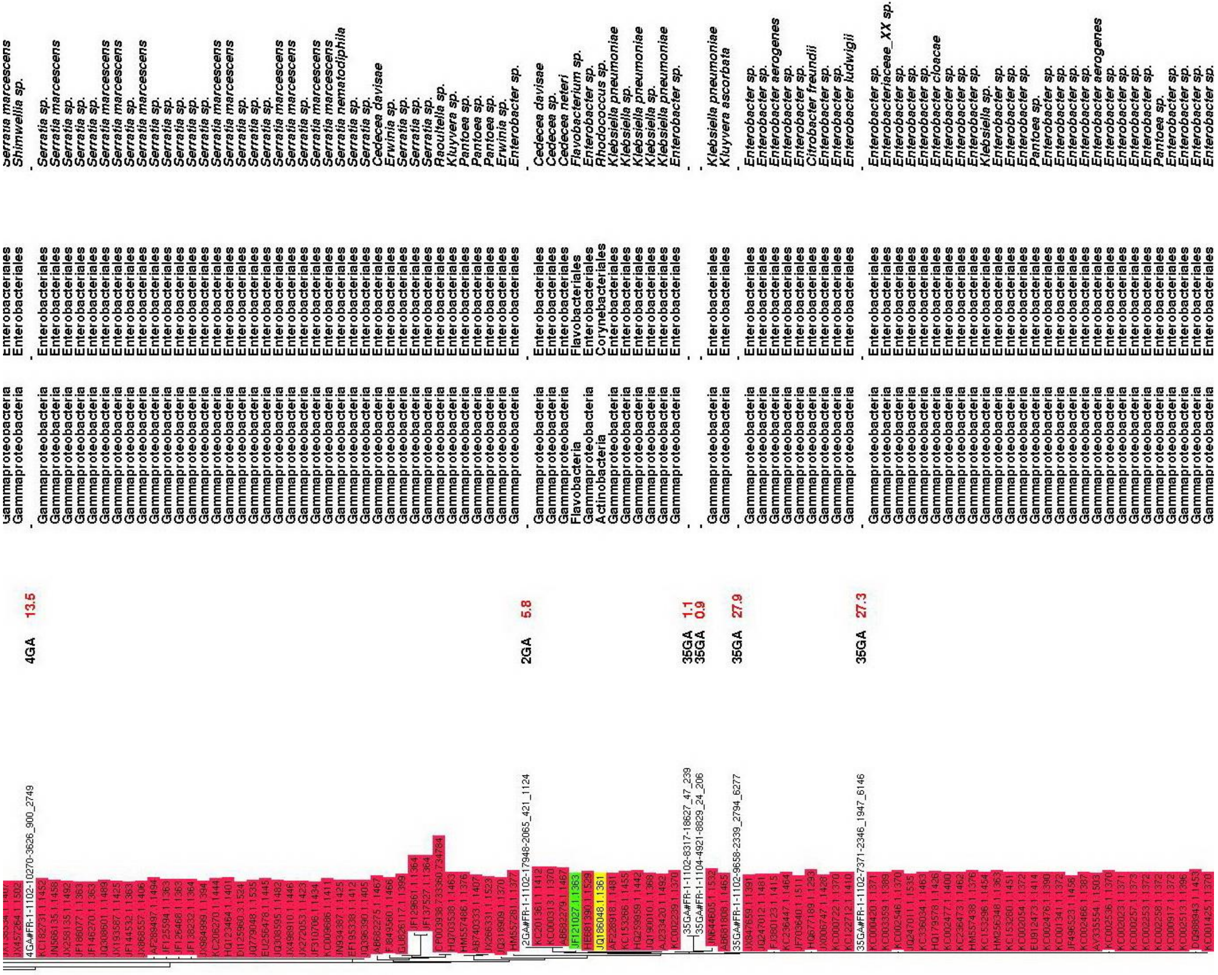

**Supplementary Figure S5D**
